# Annotation-free phenotype prediction using knowledge-augmented clustering from single-cell RNA sequencing data

**DOI:** 10.1093/bib/bbag395

**Published:** 2026-07-20

**Authors:** Janghyun Noh, Yoobin Shin, Min Kim, Minsik Oh

**Affiliations:** Department of Artificial Intelligence, Myongji University, 34 Geobukgol-ro, Seodaemun-gu, Seoul 03674, Republic of Korea; Department of Artificial Intelligence, Myongji University, 34 Geobukgol-ro, Seodaemun-gu, Seoul 03674, Republic of Korea; Department of Data Science, Myongji University, 34 Geobukgol-ro, Seodaemun-gu, Seoul 03674, Republic of Korea; Department of Artificial Intelligence, Myongji University, 34 Geobukgol-ro, Seodaemun-gu, Seoul 03674, Republic of Korea

**Keywords:** scRNA-seq data, phenotype prediction, cell clustering, single-cell foundation model, biological interpretability

## Abstract

Single-cell RNA sequencing has emerged as a transformative tool, enabling precise phenotype prediction and the detailed identification of disease-associated cell subpopulations. However, many existing computational approaches still rely on predefined cell-type annotations during model training. This dependence makes their predictive performance highly sensitive to subjective annotation quality, labeling inconsistencies, and dataset-specific biases, ultimately hindering their generalizability across diverse patient cohorts. To address these challenges, we propose scCap, an annotation-free framework that leverages knowledge-augmented clustering for robust phenotype prediction. Specifically, the framework first constructs initial clusters from raw gene expression profiles and subsequently refines them within the embedding space of a pretrained single-cell foundation model, allowing the clusters to better reflect broader biological organization while preserving fine-grained cellular heterogeneity. The resulting knowledge-augmented clusters are then integrated into a hierarchical multiple instance learning framework with dual-level attention, enabling interpretable predictions at both the cell and cluster levels. Evaluated across three public scRNA-seq datasets, scCap consistently outperforms baseline models in predictive accuracy. Furthermore, scCap identifies disease-associated subpopulations previously reported in the literature without relying on predefined cell-type annotations. These results demonstrate that scCap provides a robust and interpretable framework for annotation-free phenotype prediction.

## Introduction

Patient phenotypes can be determined by specific cell populations with abnormal functions, making their prediction a key challenge in precision medicine [1–4]. Therefore, identifying disease-associated subpopulations from single-cell data is fundamentally important, as it enables precision approaches targeting pathogenic cells and supports the discovery of reliable biomarkers [5, 6]. In this context, single-cell RNA sequencing (scRNA-seq) has emerged as a transformative technology that provides single-cell transcriptomic profiling and facilitates the characterization of disease-associated subpopulations [7, 8]. However, predictive modeling of patient phenotypes and identification of disease-associated subpopulations using scRNA-seq data remain challenging, as the high dimensionality, technical noise, and variability in cell numbers across patients hinder both predictive accuracy and biological interpretability [9].

To address these challenges, several methodological paradigms have emerged. Among them, three representative approaches can be broadly categorized as follows, although this categorization is not exhaustive. First, subpopulation-based approaches model representative cell groups to capture disease-associated heterogeneity across patients [10, 11]. Second, attention-based approaches leverage self-attention mechanisms to identify phenotype-relevant cells and prioritize informative cellular signals [12]. Third, multiple instance learning (MIL) approaches represent each patient as a bag of cells, enabling the identification of phenotype-associated subpopulations at the sample level [13–15].

Although these approaches have improved predictive accuracy and biological interpretability, many still rely on predefined cell-type annotations for training, making their performance sensitive to label quality and consistency. As reviewed by Luecken and Theis [16], standard scRNA-seq workflows cluster cells in a low-dimensional space and assign biological labels through manual inspection of cluster-specific marker genes. Because this process involves expert-driven decisions, such as marker selection, threshold definition, and reference atlas selection, annotations can vary substantially across studies. Consequently, phenotype prediction models trained on such labels may inherit this variability, introducing noise and potential bias into downstream analyses.

To reduce this reliance on subjective labeling, phenotype prediction could instead operate directly on cluster-defined subpopulations. However, conventional clustering based solely on raw gene expression or low-dimensional embeddings is highly sensitive to sparsity, technical noise, and dataset-specific variation, which can incorrectly group transcriptionally distinct cell populations [17]. Furthermore, such approaches fail to leverage the broader biological structures learned from large-scale single-cell datasets. These limitations highlight the need for biologically informed clustering strategies that provide a robust foundation for annotation-free phenotype prediction.

Motivated by this need, we propose scCap (Single-cell Knowledge-augmented Clustering for Annotation-free Phenotype Prediction). scCap leverages the generalized cell representations learned by pretrained single-cell foundation models as prior biological knowledge. These models are pretrained in a self-supervised manner to encode shared transcriptomic organization across diverse tissues and disease states [18]. To incorporate this knowledge, scCap employs a two-step clustering strategy consisting of initialization and refinement. Initial clusters are constructed from raw gene expression profiles and subsequently refined within the embedding space of a pretrained single-cell foundation model. The resulting clusters are then integrated into a hierarchical multiple instance learning framework with dual-level attention [15], aggregating information at both the cell and cluster levels for robust and interpretable phenotype prediction.

We evaluate scCap on three public scRNA-seq datasets, where it consistently outperforms existing methods in phenotype prediction while identifying disease-associated subpopulations. Together, these results establish scCap as a robust and practical framework for annotation-free phenotype prediction grounded in knowledge-augmented clustering.

## Related work

### Subpopulation-based approaches

Early studies modeled cellular subpopulations within patient samples to capture disease-relevant structures. CloudPred [10], for instance, approximates cell distributions using a Gaussian mixture model and leverages component prevalence for phenotype prediction. ProtoCell4P [11] instead learns representative prototypes in a latent space and assigns cells accordingly, enabling prototype-based prediction. While computationally efficient, these methods rely on predefined or assumed subpopulation structures, which may inadequately reflect the biological heterogeneity of real-world datasets and remain sensitive to data quality.

### Attention-based approaches

Attention-based models have also been developed for phenotype prediction from scRNA-seq data. ScRAT [12] employs a Transformer-based self-attention mechanism to identify influential cells and incorporates mixup augmentation to address small-sample limitations. These approaches remove the need for explicit annotations and enable data-driven identification of critical cells. However, they still depend on subpopulation information during augmentation, and interpretability remains constrained, as attention weights alone do not necessarily provide biologically grounded explanations.

### Multiple instance learning-based approaches

The bag-of-cells nature of scRNA-seq data naturally motivates the application of MIL, where each patient is represented as an unordered collection of cells. Ilse *et al*. [19] demonstrated that attention-based MIL improves predictive performance while offering instance-level interpretability. Building on this framework, MixMIL [13] incorporated a generalized linear mixed model to explicitly account for sample heterogeneity and enhance robustness. More recently, scMILD [14] introduced a weakly supervised dual-branch design that jointly performs sample-level classification and subpopulation discovery. Along a complementary direction, HA [15] proposed dual-level attention across cells and cell types, leveraging hierarchical annotation information to further improve performance and interpretability. Nevertheless, reliance on predefined annotations limits robustness, as model performance becomes sensitive to annotation quality.

## Methods

### Overview

**Figure 1:**
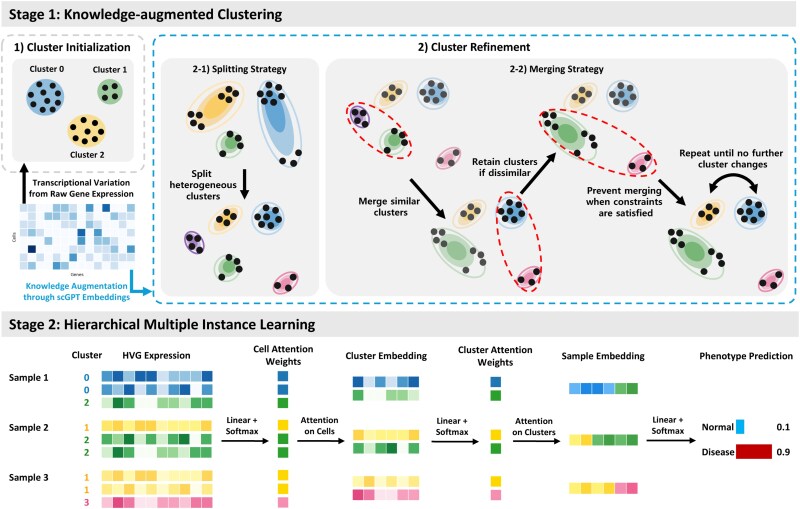
Overview of scCap, where Stage 1 performs knowledge-augmented clustering by initializing clusters in the raw gene expression space and refining them in the scGPT embedding space to integrate biologically informed representations, and Stage 2 incorporates the refined clusters into a hierarchical multiple instance learning framework with dual-level attention, aggregating information at both the cell and cluster levels for robust phenotype prediction.

In this section, we introduce scCap, a two-stage framework designed for annotation-free phenotype prediction. As illustrated in Fig. 1, scCap first constructs clusters through knowledge-augmented clustering and then integrates these clusters into a hierarchical MIL framework for robust and interpretable phenotype prediction.

The first stage, knowledge-augmented clustering, constructs clusters through a two-step procedure of cluster initialization and refinement. During initialization, clusters are derived from raw gene expression to capture local transcriptional variation underlying fine-grained cellular heterogeneity. In the refinement step, these clusters are optimized in the embedding space of scGPT [20], a pretrained single-cell foundation model that encodes biological structure learned from large-scale single-cell datasets. By incorporating this knowledge, scCap refines cluster boundaries to better reflect global biological organization while preserving fine-grained heterogeneity. To achieve this, we employ a split–merge strategy that divides heterogeneous clusters and iteratively merges biologically similar ones, resulting in stable and biologically coherent cluster structures.

In the second stage, hierarchical MIL, incorporates the refined clusters into a dual-level attention architecture that aggregates information at both the cell and cluster levels [15]. This hierarchical design enables robust and interpretable phenotype prediction, while facilitating the identification of biologically meaningful disease-associated subpopulations.

### Stage 1: Knowledge-augmented clustering

#### Cluster initialization

We first construct initial clusters from raw gene expression profiles to capture local transcriptional variation within each patient sample. The resulting clusters reflect local similarity patterns in the transcriptomic space and serve as the basis for subsequent refinement.

Specifically, following standard Scanpy-based single-cell analysis procedures [21], we first normalize and log-transform the expression data, and then perform principal component analysis (PCA; top 50 components). Based on the PCA representation, we construct a $k$-nearest-neighbors graph ($k{=}15$) and apply Leiden clustering [22] with resolution $1.0$ to obtain initial cell groups.

We do not apply explicit dropout correction or imputation during this process, consistent with prior clustering studies such as He *et al*. [17], where clustering is performed directly on normalized expression profiles without explicit imputation. We adopt this strategy because imputation procedures can alter local transcriptional relationships and potentially influence downstream clustering behavior.

#### Cluster refinement

To mitigate the noise inherent in raw gene expression and leverage broader biological priors, the refinement step implements knowledge augmentation by re-aligning the initial clusters within the scGPT embedding space, a biologically informed representation learned from large-scale single-cell data. Operating in this representation space reduces dataset-specific variation while improving the biological coherence of the resulting clusters.

To refine cluster structure, scCap adopts a split–merge strategy in the scGPT embedding space. Intuitively, clusters with high internal heterogeneity are split to separate distinct subpopulations, whereas clusters that are highly similar in the embedding space are merged to recover coherent biological groups and reduce redundancy. This design encourages clusters to be both internally coherent and well separated, allowing the resulting groups to better reflect the biological knowledge learned by the pretrained scGPT model.

To determine whether a cluster should be split, scCap evaluates each cluster using two complementary distance measures: an intra-cluster distance quantifying within-cluster dispersion and an inter-cluster distance measuring global separation between clusters.

For each cluster $C$ obtained during the initialization step, the intra-cluster distance is defined as 


(1) \begin{align*}& d_{\mathrm{intra}}(C) = \frac{2}{|C|(|C|-1)} \sum_{i<j,\, i,j \in C} \| x_{i} - x_{j} \|_{2},\end{align*}

where $x_{i}$ and $x_{j}$ denote the scGPT embeddings of cells $i$ and $j$ in cluster $C$. Using scGPT embeddings provides a biologically informed representation that reflects broader biological organization beyond local transcriptional variation.

The inter-cluster distance is defined as the average distance between the centroids of different clusters: 


(2) \begin{align*}& d_{\mathrm{inter}} = \frac{2}{K(K-1)} \sum_{a<b} \| c_{a} - c_{b} \|_{2},\end{align*}

where $c_{a}$ and $c_{b}$ are the centroids of clusters $a$ and $b$, and $K$ denotes the total number of clusters. Each centroid is computed as 


(3) \begin{align*}& c_{a} = \frac{1}{|C_{a}|} \sum_{i \in C_{a}} x_{i},\end{align*}

where $x_{i}$ represents the scGPT-based embedding of cell $i$.

We then quantify cluster compactness and separation to determine whether a cluster should be split. A cluster is considered overly dispersed if 


(4) \begin{align*}& d_{\mathrm{intra}}(C)> \tfrac{1}{2} d_{\mathrm{inter}},\end{align*}

in which case it is subdivided into two subclusters using the K-medoids algorithm [23]. This criterion indicates that the within-cluster variation exceeds half of the average inter-cluster separation, suggesting that the cluster lacks internal coherence in the knowledge-augmented space. By selectively splitting only highly heterogeneous clusters, scCap preserves compact and biologically coherent structures while avoiding unnecessary fragmentation.

Subsequently, scCap merges highly similar clusters to recover broader biological organization and reduce redundancy. Cluster similarity is evaluated based on centroid proximity in the scGPT embedding space, where biologically related populations are expected to reside in adjacent regions. Through this sequential split–merge refinement, scCap produces stable cluster structures aligned with biologically informed representations while preserving fine-grained cellular heterogeneity.

Formally, for each cluster $C$ obtained after splitting, the intra-cluster distance relative to its centroid $c_{C}$ is defined as 


(5) \begin{align*}& d_{\mathrm{intra}}(C) = \frac{1}{|C|} \sum_{x \in C} \| x - c_{C} \|_{2},\end{align*}

and the inter-cluster distance between two clusters $(C_{a}, C_{b})$ is defined as 


(6) \begin{align*}& d_{\mathrm{inter}}(C_{a},C_{b}) = \| c_{a} - c_{b} \|_{2},\end{align*}

where $c_{a}$ and $c_{b}$ denote the centroids of $C_{a}$ and $C_{b}$, respectively.

However, relying solely on centroid proximity may result in inappropriate merging when clusters exhibit high internal dispersion. To mitigate this issue, following He *et al*. [17], we introduce a correction weight that penalizes clusters with large intra-cluster variability, thereby balancing centroid proximity and compactness.

The global average intra-cluster distance is computed as 


(7) \begin{align*}& \bar d_{\mathrm{intra}} = \frac{1}{K} \sum_{k=1}^{K} d_{\mathrm{intra}}(C_{k}),\end{align*}

providing a global baseline for assessing compactness. The correction weight is defined as 


(8) \begin{align*}& w_{ab} = \frac{\bar d_{\mathrm{intra}}} {\tfrac{1}{2}\big(d_{\mathrm{intra}}(C_{a})+d_{\mathrm{intra}}(C_{b})\big)},\end{align*}

which reduces the likelihood of merging clusters that are internally heterogeneous.

The weighted inter-cluster distance is then defined as 


(9) \begin{align*}& d^{w}(C_{a},C_{b}) = w_{ab}\, d_{\mathrm{inter}}(C_{a},C_{b}),\end{align*}

and its global average as 


(10) \begin{align*}& \bar d^{w}_{\mathrm{inter}} = \frac{2}{K(K-1)} \sum_{a<b} d^{w}(C_{a},C_{b}),\end{align*}

which serves as a dynamic threshold for merge decisions.

Two clusters are considered mergeable if 


(11) \begin{align*}& d^{w}(C_{a},C_{b}) < \frac{\bar d^{w}_{\mathrm{inter}}}{\rho},\end{align*}

where $\rho $ controls the strictness of the merging criterion. Larger $\rho $ values enforce more conservative merging, whereas smaller values permit more aggressive consolidation. In our experiments, we set $\rho = 2.0$. We further provide a sensitivity analysis of this parameter in Section S6.3 of the supplementary material, demonstrating that the method remains robust across a range of values.

To avoid excessive loss of granularity, we impose an additional constraint on the size of merged clusters as 


(12) \begin{align*}& |C_{a}| + |C_{b}| \le \tfrac{1}{2}N,\end{align*}

where $N$ denotes the total number of cells.

Merging proceeds iteratively by selecting the closest eligible pair at each step and updating centroids and distances after every merge. This adaptive split–merge refinement balances local transcriptional variation with global biological organization, yielding biologically coherent and granularity-preserving clusters for downstream phenotype prediction.

### Stage 2: Hierarchical multiple instance learning

Using the refined clusters, scCap performs phenotype prediction within a hierarchical MIL framework. Each patient is represented as a bag of clusters, and each cluster as a bag of cells. A dual-level attention mechanism first aggregates cell representations into cluster-level embeddings and then aggregates cluster embeddings into a patient-level representation.

This hierarchical design captures biological heterogeneity at both the cellular and cluster levels, while enhancing interpretability by identifying the most informative cells and clusters. To preserve local transcriptional structure and improve computational efficiency, we restrict the input to the top 3000 highly variable genes (HVGs), retaining biologically meaningful variation while reducing dimensionality.

#### Cell-to-cluster aggregation

Before aggregation, each cell feature vector is transformed into a lower-dimensional representation to facilitate effective cluster-level integration. Let $x_{ij} \in \mathbb{R}^{m}$ denote the HVG-based expression vector of cell $j$ in cluster $i$, where $m = 3000$ corresponds to the number of HVGs. Each cell is mapped to a lower-dimensional representation through a neural network layer: 


(13) \begin{align*}& h_{ij} = \phi(W_{x} x_{ij} + b_{x}),\end{align*}

where $W_{x} \in \mathbb{R}^{d \times m}$ and $b_{x} \in \mathbb{R}^{d}$ are trainable parameters, and $\phi (\cdot )$ denotes a nonlinear activation function such as ReLU.

We then apply an attention-based aggregation mechanism, similar to prior attention-based MIL approaches [19], to obtain a cluster-level embedding from cell representations, where attention weights are computed using shared learnable parameters. For each cell $j$ in cluster $i$, an attention score $e_{ij}$ and corresponding weight $\alpha _{ij}$ are computed as 


(14) \begin{align*}& e_{ij} = w^\top h_{ij} + b, \quad \alpha_{ij} = \frac{\exp(e_{ij})}{\sum_{j^{\prime}=1}^{n_{i}} \exp(e_{ij^{\prime}})},\end{align*}

where $w \in \mathbb{R}^{d}$ and $b \in \mathbb{R}$ are shared learnable parameters, and $n_{i}$ denotes the number of cells in cluster $i$. The cluster-level representation is then obtained as 


(15) \begin{align*}& h_{i} = \sum_{j=1}^{n_{i}} \alpha_{ij} h_{ij}.\end{align*}

#### Cluster-to-patient aggregation

At the second level, cluster representations are aggregated into a patient-level representation. For each cluster $i$, an attention score $e_{i}$ and weight $\beta _{i}$ are computed as 


(16) \begin{align*}& e_{i} = v^\top h_{i} + b_{0}, \quad \beta_{i} = \frac{\exp(e_{i})}{\sum_{i^{\prime}=1}^{K} \exp(e_{i^{\prime}})},\end{align*}

where $v \in \mathbb{R}^{d}$ and $b_{0} \in \mathbb{R}$ are trainable parameters, and $K$ represents the number of clusters within a patient. The final patient-level representation is given by 


(17) \begin{align*}& h = \sum_{i=1}^{K} \beta_{i} h_{i}.\end{align*}

#### Prediction layer

After obtaining the patient-level representation $h$ through hierarchical aggregation, this representation is fed into a prediction layer to compute phenotype probabilities. For binary classification tasks, a sigmoid function is applied: 


(18) \begin{align*}& p = \sigma(w_{\mathrm{cls}}^\top h + b_{\mathrm{cls}}),\end{align*}

where $w_{\mathrm{cls}} \in \mathbb{R}^{d}$ and $b_{\mathrm{cls}} \in \mathbb{R}$ are trainable parameters. For multi-class classification with $C$ classes, a softmax function is used: 


(19) \begin{align*}& p = \mathrm{softmax}(W_{\mathrm{cls}} h + b_{\mathrm{cls}}),\end{align*}

where $W_{\mathrm{cls}} \in \mathbb{R}^{C \times d}$ and $b_{\mathrm{cls}} \in \mathbb{R}^{C}$ are trainable parameters.

Model optimization is performed by minimizing the cross-entropy loss between the predicted probabilities $p$ and the ground-truth phenotype labels. Given $S$ patient samples and $C$ classes, the loss function is defined as 


(20) \begin{align*}& \mathcal{L} = - \sum_{s=1}^{S} \sum_{c=1}^{C} y_{s,c} \log(p_{s,c}),\end{align*}

where $y_{s,c} \in \{0,1\}$ indicates whether sample $s$ belongs to class $c$, and $p_{s,c}$ denotes the corresponding predicted probability.

We optimize the model using the Adam optimizer, with hyperparameters including the learning rate, weight decay, and dropout rate tuned using Optuna within each training split. For each outer fold, the best configuration is selected based on validation performance and used to retrain the model. Detailed optimization settings and full hyperparameter configurations are provided in Section S1 of the supplementary material.

#### Interpretability of the model

Since both aggregation steps employ attention and the prediction layer is linear, the final logits can be decomposed into weighted contributions from individual cells and clusters. Using the hierarchical attention weights $\gamma _{ij} = \beta _{i} \alpha _{ij}$, the patient representation can be expressed as 


(21) \begin{align*}& h = \sum_{i=1}^{K} \sum_{j=1}^{n_{i}} \gamma_{ij} h_{ij}, \quad \mathrm{with} \quad \sum_{i=1}^{K}\sum_{j=1}^{n_{i}} \gamma_{ij} = 1.\end{align*}

This formulation makes explicit the contribution of each cell (and its cluster) to the final prediction, which directly extends to the logit for class $c$: 


(22) \begin{align*}& z_{c} = W_{c} h + b_{c} = \sum_{i=1}^{K} \sum_{j=1}^{n_{i}} \gamma_{ij}\,(W_{c} h_{ij}) + b_{c},\end{align*}

showing that each logit is a weighted sum of cell-level contributions.

To further quantify the contribution of each cluster, we compute an importance score that reflects its overall impact on predicting a given class. Let $\ell ^{(c)}_{pi}$ denote the logit contribution of cluster $i$ in patient $p$ for class $c$, obtained by aggregating the attention-weighted cell representations within cluster $i$. The importance score of cluster $i$ for class $c$ is defined as 


(23) \begin{align*}& \kappa_{i}^{(c)} = \frac{1}{|P_{c}|} \sum_{p \in P_{c}} \ell^{(c)}_{pi} - \frac{1}{|P_{\neg c}|} \sum_{p \in P_{\neg c}} \ell^{(c)}_{pi},\end{align*}

where $P_{c}$ is the set of patients labeled as class $c$, and $P_{\neg c}$ is the set of patients not labeled as class $c$. A higher $\kappa _{i}^{(c)}$ indicates that cluster $i$ contributes more strongly to distinguishing class $c$ from the others.

From a biological perspective, clusters with high importance scores correspond to subpopulations strongly associated with the disease phenotype. These subpopulations provide interpretable evidence of the cellular groups driving the predicted phenotype and may reveal biologically relevant populations with potential diagnostic or therapeutic significance.

## Experiments and results

### Datasets

We evaluate scCap against baseline models on three public datasets: COVID [24], Cardio [25], and Kidney [26]. These datasets are representative benchmark datasets commonly used in prior phenotype prediction studies using scRNA-seq data. A summary of the datasets is provided in Table 1.

**Table 1:** Summary of the datasets used in this study.

Dataset	Patients	Classes	Cell Types	Cells	Genes
COVID	50	Normal: 15 Infection: 35	18	26 947	28 696
Cardio	42	Normal: 16 HCM: 15 DCM: 11	13	592 689	32 151
Kidney	77	Normal: 26 CKD: 37 AKF: 14	25	225 177	29 125

The COVID dataset comprises scRNA-seq profiles from patients with COVID-19 and healthy controls. Following prior work [15], the long-COVID and respiratory failure groups were excluded due to limited sample sizes, resulting in a binary classification task. The Cardio dataset consists of single-nucleus RNA-seq profiles from patients with dilated cardiomyopathy (DCM), hypertrophic cardiomyopathy (HCM), and healthy controls, forming a three-class classification problem. Although scCap was originally developed for scRNA-seq analysis, the proposed framework operates on transcriptomic expression representations that are also available in snRNA-seq data. In addition, the same Cardio dataset has also been used in prior phenotype prediction studies [11, 15]. We note that potential differences between scRNA-seq and snRNA-seq (e.g. nuclear transcript bias) may affect expression profiles, but do not limit the applicability of the proposed framework. The Kidney dataset includes scRNA-seq profiles from patients with acute kidney failure (AKF), chronic kidney disease (CKD), and healthy controls, also constituting a three-class task.

For all datasets, we applied a unified preprocessing pipeline following prior studies [15] to ensure fair comparison: (i) removing genes expressed in fewer than five cells, (ii) normalizing total counts to $10^{4}$, and (iii) applying log-transformation.

We further extracted cell embeddings from a pretrained scGPT model [20] for knowledge-augmented clustering. Specifically, we used the whole-human model pretrained on 33 million human single-cell profiles to encode each cell into a 512D embedding. None of the datasets used in this study were included in the scGPT pretraining corpus, thereby preventing data leakage.

Finally, to assess the sensitivity of annotation-based models to label quality, we generated additional computational annotations using SingleR [27] with the Human Primary Cell Atlas as reference [28]. These automatically derived labels served as an alternative annotation source for annotation-based baselines, enabling direct comparison with manually curated labels.

### Baselines and evaluation

We compare scCap with representative baseline models for phenotype prediction from single-cell data, spanning the three methodological paradigms discussed in Related Work: CloudPred [10], which models subpopulation structure using a Gaussian mixture model; ProtoCell4P [11], which learns prototype representations of informative cell subsets; ScRAT [12], a Transformer-based framework leveraging self-attention to identify critical cells; and HA [15], which extends MIL with dual-level attention across cells and cell types. These baselines are representative recent frameworks for patient-level phenotype prediction from single-cell transcriptomic data and are commonly used in related studies.

Predictive performance is primarily evaluated using the area under the receiver operating characteristic curve (AUROC) and the area under the precision–recall curve (AUPRC). AUROC is computed directly for binary tasks and under a one-versus-rest scheme for multi-class settings. AUPRC provides complementary insight, particularly in the presence of class imbalance. We additionally report accuracy, precision, recall, and F1 score for completeness.

To ensure fair and reliable evaluation, we adopt nested cross-validation with five outer folds and five inner folds, repeated five times to reduce variance due to data partitioning. Hyperparameter tuning is conducted within inner folds, while performance is reported on outer folds. All models are evaluated on identical predefined splits to eliminate variability arising from different train–test partitions. We provide model-specific hyperparameters and optimization details in Section S1 of the supplementary material.

### Results

#### Performance comparison with baseline models

Table 2 summarizes the predictive performance of scCap and baseline models across three public datasets [24–26], evaluated using AUROC and AUPRC. We report additional metrics, including accuracy, precision, recall, and F1 score, in Section S2 of the supplementary material.

**Table 2:** Comparison of AUROC and AUPRC (Mean $\pm $ Std) across three datasets.

Metric	Method	COVID	Cardio	Kidney
AUROC	CloudPred	0.89 $\pm $ 0.03	0.89 $\pm $ 0.01	0.90 $\pm $ 0.02
	ProtoCell4P	0.80 $\pm $ 0.07	0.88 $\pm $ 0.02	0.83 $\pm $ 0.05
	ScRAT	0.86 $\pm $ 0.21	0.93 $\pm $ 0.10	0.88 $\pm $ 0.10
	HA	0.89 $\pm $ 0.05	0.87 $\pm $ 0.02	0.92 $\pm $ 0.01
	scCap	**0.93 $\pm $ 0.03**	**0.97 $\pm $ 0.01**	**0.94 $\pm $ 0.02**
AUPRC	CloudPred	0.95 $\pm $ 0.01	0.81 $\pm $ 0.03	0.87 $\pm $ 0.02
	ProtoCell4P	0.65 $\pm $ 0.16	0.77 $\pm $ 0.03	0.71 $\pm $ 0.07
	ScRAT	0.93 $\pm $ 0.10	0.90 $\pm $ 0.13	0.82 $\pm $ 0.13
	HA	0.95 $\pm $ 0.02	0.82 $\pm $ 0.02	0.86 $\pm $ 0.02
	scCap	**0.97 $\pm $ 0.01**	**0.96 $\pm $ 0.01**	**0.91 $\pm $ 0.04**

Note: CloudPred and scCap do not rely on annotation information and are evaluated under the *Free* condition. Bold values indicate the best performance for each dataset and evaluation metric.

For annotation-based baselines (ProtoCell4P, ScRAT, and HA), experiments were conducted using manually curated labels (*Manual*), whereas annotation-free methods (CloudPred and scCap) were evaluated without external label information. Additional comparisons across different annotation conditions, including manual and SingleR-based annotations, are provided in the Section S3 of the supplementary material. These results further indicate that the performance of annotation-based methods can vary depending on the annotation source and quality.

As shown in Table 2, scCap consistently achieves the highest AUROC and AUPRC across all three datasets, outperforming both annotation-based and annotation-free baselines. Compared with the annotation-free CloudPred, scCap improves AUROC by 0.04 (COVID), 0.08 (Cardio), and 0.04 (Kidney), with corresponding gains in AUPRC.

Collectively, these results demonstrate that knowledge-augmented clustering enables robust and competitive annotation-free phenotype prediction across diverse biological contexts.

### Ablation study on knowledge-augmented clustering

To systematically assess how knowledge-augmented clustering improves predictive performance, we conduct an ablation study analyzing the contribution of its core components. Specifically, we evaluate the impact of (i) cluster refinement, (ii) biological knowledge integration, and (iii) the merging constraint applied during clustering on downstream phenotype prediction.

#### Effect of refinement

We first evaluate the effect of the refinement step by comparing the full model with a *No-Refinement* variant, where the split–merge refinement step is omitted and the initial clusters are directly used.

The refinement step applies a split–merge strategy to reorganize initial clusters. Rather than merely reassigning cells, refinement explicitly reduces intra-cluster heterogeneity while preserving biologically coherent relationships. By splitting heterogeneous clusters and merging biologically similar ones, this procedure yields a more structured and internally consistent clustering configuration.

**Figure 2:**
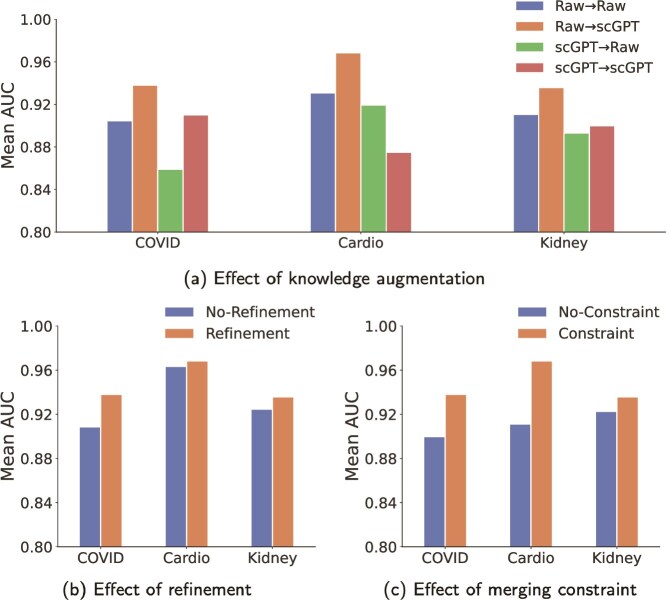
Ablation study of scCap. (a) Effect of biological knowledge integration: each configuration indicates the feature space used for initial clustering and refinement, respectively. Specifically, *Raw*$\rightarrow $*Raw* uses gene expression for both steps, *Raw*$\rightarrow $*scGPT* performs initial clustering on raw expression and refinement in the scGPT embedding space, *scGPT*$\rightarrow $*Raw* performs the reverse, and *scGPT*$\rightarrow $*scGPT* uses scGPT embeddings for both steps. Among these, *Raw*$\rightarrow $*scGPT* achieves the highest AUROC across datasets. (b) Effect of refinement: comparison between models without refinement (*No-Refinement*) and with the split–merge refinement strategy (*Refinement*), showing that refinement consistently improves predictive performance. (c) Effect of merging constraint: comparison between models without the merging constraint (*No-Constraint*) and with the constraint (*Constraint*), demonstrating that preventing excessive merging leads to improved phenotype prediction.

As shown in Fig. 2b, incorporating refinement consistently improves AUROC across all datasets. These findings suggest that reorganizing clusters to better reflect biological structure enhances the quality of cluster-level representations, thereby improving phenotype prediction performance.

#### Effect of biological knowledge integration

We evaluate the effect of biological knowledge integration by comparing configurations that differ in the representation space used for cluster initialization and refinement, where each setting is denoted as (initialization space$\rightarrow $refinement space).

As illustrated in Fig. 2a, the *Raw*$\rightarrow $*scGPT* configuration consistently achieves the highest AUROC across all datasets.

This result highlights the benefit of performing refinement in a biologically informed embedding space. Initializing clusters in the raw gene expression space preserves fine-grained transcriptional variability, while refinement in the scGPT embedding reorganizes clusters according to biologically aligned structure. The complementary strengths of these representations enable the hybrid strategy to produce more discriminative cluster-level features.

In contrast, using only raw expression or only pretrained embeddings results in inferior performance, indicating that neither representation alone sufficiently captures both transcriptional detail and biological structure. Furthermore, reversing the order (*scGPT*$\rightarrow $*Raw*) degrades performance, suggesting that projecting clusters back into the raw space disrupts the biologically aligned organization established in the embedding space.

#### Effect of merging constraint

We examine the impact of the merging constraint on phenotype prediction performance by comparing the full model with the *No-Constraint* variant, where the constraint designed to prevent excessive cluster merging is removed.

As shown in Fig. 2c, enforcing this constraint consistently improves AUROC across datasets.

By restricting excessive merging of heterogeneous clusters, the constraint preserves biologically distinct subpopulations and stabilizes the cluster structure. This stabilization leads to more reliable cluster-level representations and contributes to improved phenotype prediction performance.

### Interpretability of scCap predictions

To identify subpopulations contributing to scCap’s predictions, we quantified cluster-level importance scores across all constructed clusters. We present the COVID dataset as a representative example, while corresponding analyses for the Cardio and Kidney datasets are provided in Section S4 of the supplementary material.

**Figure 3:**
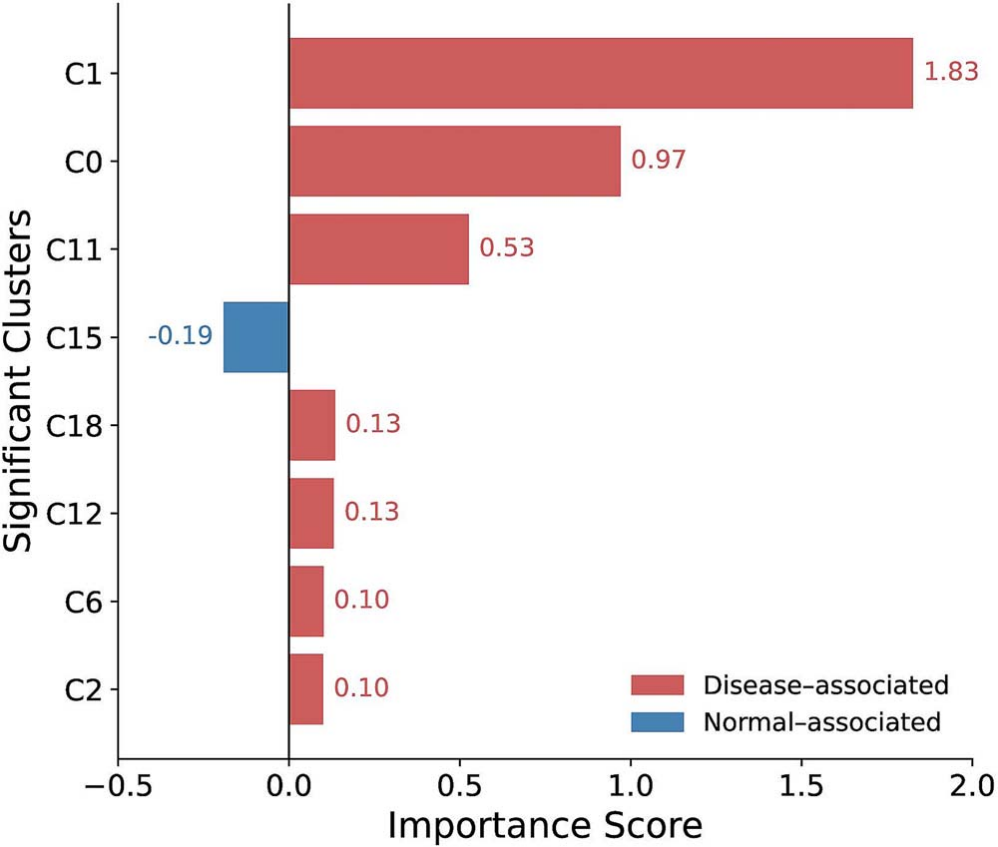
Importance scores of significant subpopulations identified in the COVID dataset. The positive values indicate stronger associations with the disease state, whereas the negative values indicate associations with the healthy state.

Clusters with absolute importance scores $\geq 0.1$ were defined as significant subpopulations. Positive scores indicate stronger associations with the disease state, whereas negative scores reflect associations with the healthy state. The importance scores of these subpopulations are shown in Fig. 3, illustrating their relative contributions to the model’s predictive outcome.

**Figure 4:**
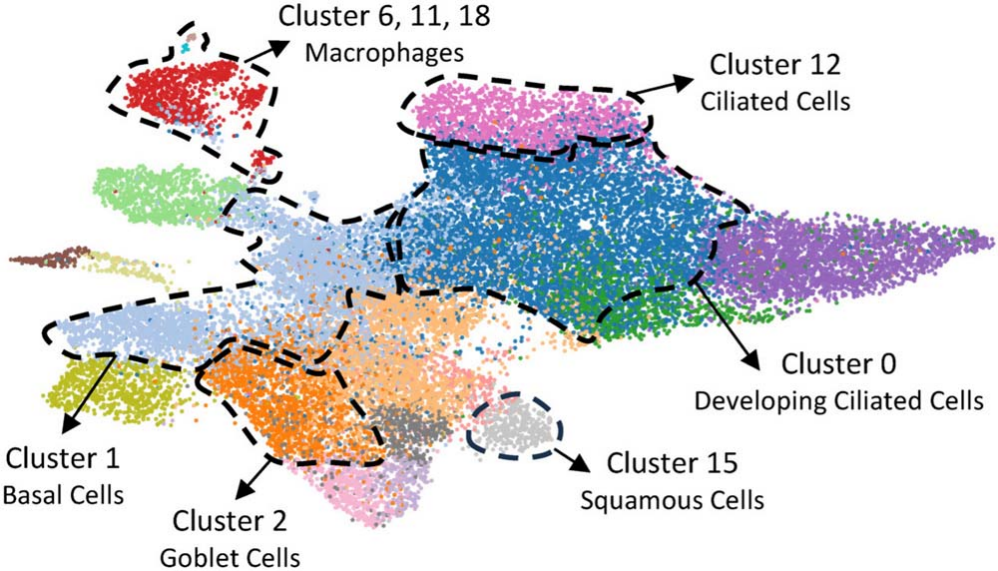
UMAP visualization of all clusters in the COVID dataset. The dashed outlines highlight significant subpopulations and their inferred cell-type identities. The spatial organization indicates that biologically related epithelial states are grouped into coherent clusters.

**Figure 5:**
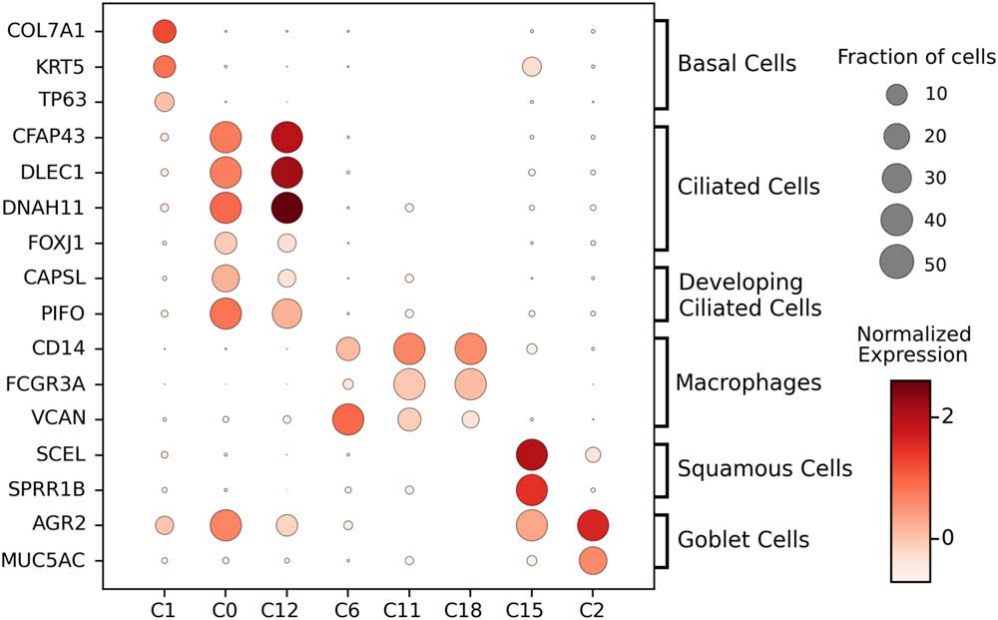
Canonical marker expression patterns used to infer the putative cell-type identities of significant subpopulations in the COVID dataset. Dot size represents the proportion of expressing cells, and color intensity indicates average expression level.

To further examine the structural organization of the identified subpopulations, we visualized all clusters in UMAP space (Fig. 4), highlighting significant clusters with dashed boundaries and annotating their putative cell-type identities. These identities were inferred from cluster-specific gene expression patterns and interpreted using canonical marker genes curated from prior literature, as summarized in Fig. 5. To support this interpretation, we performed differential gene expression analysis using the Wilcoxon rank-sum test implemented in Scanpy, a widely used method for single-cell RNA-seq analysis, which identified key markers such as *KRT5*, *FOXJ1*, and *MUC5AC* among the top differentially expressed genes in their respective clusters.

Notably, cluster 0 (C0) and cluster 12 (C12) were located in close proximity in UMAP space and both exhibited canonical ciliated cell signatures, with shared enrichment of mature ciliated markers including *CFAP43*, *DLEC1*, *DNAH11*, and *FOXJ1*. Despite this shared lineage identity, C0 showed elevated expression of *CAPSL* and *PIFO*, which are associated with developing ciliated cells [24], suggesting a less differentiated ciliated state than C12.

Importantly, this distinction was identified without reliance on predefined cell-type annotations. These findings demonstrate that the knowledge-augmented clustering framework captures subtle epithelial differentiation heterogeneity and integrates this knowledge-guided structure into phenotype prediction.

### Biological interpretation of significant subpopulations

Previous studies have identified the airway epithelium as a primary site of SARS-CoV-2 infection [29]. Within this compartment, basal cells serve as multipotent progenitors that restore epithelial integrity by differentiating into ciliated and goblet (secretory) lineages during regeneration [30]. Viral exposure has further been shown to enhance differentiation signals toward the ciliated lineage [31]. Collectively, these findings position the basal-to-ciliated differentiation axis as a biologically relevant framework for understanding epithelial remodeling in COVID-19.

**Figure 6:**
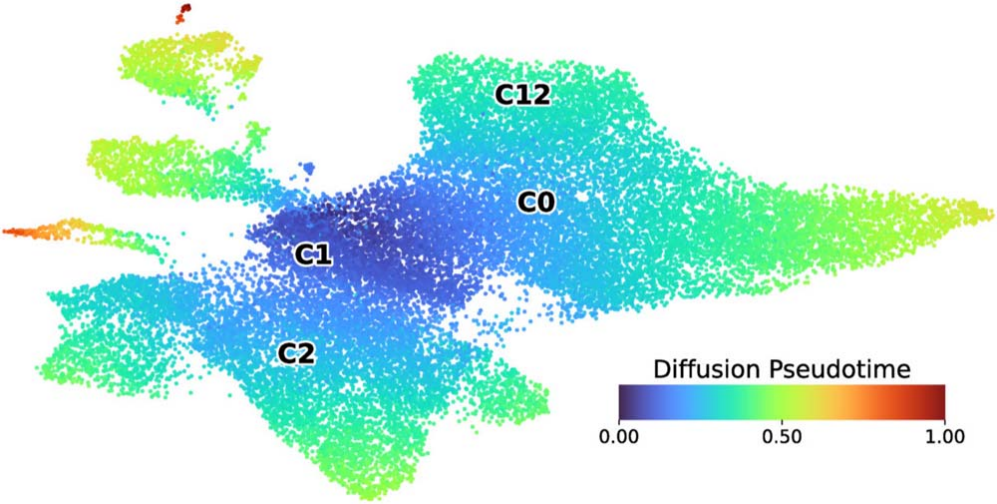
DPT analysis of epithelial subpopulations in the COVID dataset. The trajectory reveals a continuous progression from basal to developing and mature ciliated states, with branching toward a goblet-like lineage. Clusters C1 and C0 occupy distinct positions along the basal-to-ciliated differentiation axis, consistent with their inferred basal and developing ciliated identities.

Consistent with this framework, several significant subpopulations identified by scCap aligned with epithelial lineage hierarchies. Diffusion pseudotime (DPT) analysis [32] revealed a continuous trajectory originating from the inferred basal-lineage cluster C1, progressing toward C0 and C12 along a ciliated lineage axis, and branching toward C2, corresponding to a goblet-like state. These findings indicate that the identified subpopulations recapitulate established basal-driven epithelial differentiation programs (Fig. 6).

**Figure 7:**
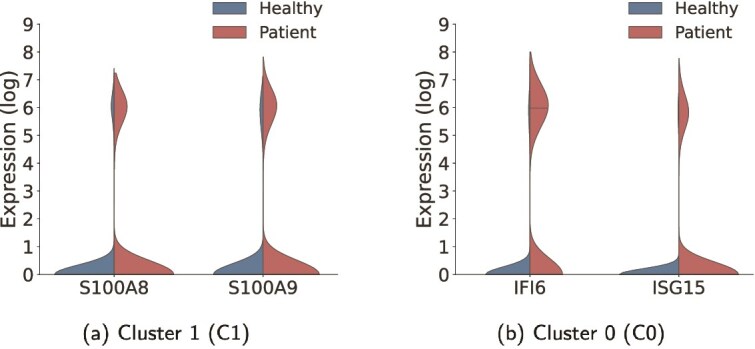
Differential gene expression in the top disease-associated subpopulations identified by scCap in the COVID dataset. (a) C1 shows upregulation of *S100A8* and *S100A9* in COVID-19 patients compared to healthy controls, consistent with calprotectin-associated inflammatory responses. (b) C0 exhibits increased expression of ISGs (*IFI6*, *ISG15*) in COVID-19 patients, indicating activation of antiviral transcriptional programs in a developing ciliated state. These patterns support the biological relevance of the subpopulations prioritized by scCap.

Notably, the two subpopulations assigned the highest predictive importance by scCap, C1 and C0, corresponded to distinct stages along this basal-driven axis. Previous studies have shown that SARS-CoV-2 infection disrupts basal cell homeostasis and perturbs epithelial regenerative dynamics [33, 34]. Consistent with these observations, the inferred basal-lineage cluster C1 exhibited significantly increased expression of *S100A8* and *S100A9* in COVID-19 patients compared with healthy controls (Fig. 7a). As components of the calprotectin complex, these genes are associated with inflammatory responses and severe COVID-19 progression [35]. Together, these findings suggest that C1 represents a regenerating basal epithelial state characterized by inflammatory activation.

Similarly, the second highly ranked cluster, C0, showed elevated expression of interferon-stimulated genes (ISGs), including *IFI6* and *ISG15*, in COVID-19 patients (Fig. 7b). Previous work has documented induction of ISGs and dynamic epithelial state transitions during SARS-CoV-2 infection [36]. The enrichment of antiviral transcriptional programs in C0 therefore supports the presence of epithelial-intrinsic interferon responses within a developing ciliated state.

This suggests a transition from inflammatory activation in basal-like states (C1) to interferon-mediated antiviral responses in developing ciliated states (C0).

Taken together, these findings indicate that the subpopulations prioritized by knowledge-augmented clustering are not arbitrary groupings but correspond to biologically coherent, disease-relevant epithelial states. By capturing both regenerative–inflammatory and antiviral programs along the basal-to-ciliated axis, the inferred trajectory organizes subpopulations in a manner consistent with established COVID-19 epithelial remodeling dynamics, characterized by inflammatory activation (e.g. S100A8/A9 [35]), interferon-mediated antiviral responses (e.g. IFI6, ISG15 [36]), and epithelial regeneration following viral-induced damage [30, 31].

## Discussion

In this study, we introduced scCap, an annotation-free framework for phenotype prediction from scRNA-seq data. Instead of relying on predefined cell-type annotations, scCap performs phenotype prediction based on knowledge-augmented subpopulations constructed by integrating fine-grained clustering with biological knowledge encoded in pretrained single-cell foundation model representations. These subpopulations preserve cellular heterogeneity while capturing broader transcriptomic organization, providing robust and interpretable units for downstream phenotype prediction.

Across three public datasets [24–26], scCap consistently improved predictive performance over baseline models while maintaining biological interpretability. Through knowledge-augmented clustering, transcriptionally similar cells are organized into structured subpopulations, and the hierarchical MIL framework highlights disease-associated clusters whose transcriptional programs align with mechanisms reported in prior studies. These findings suggest that biologically informed clustering reduces dependence on predefined annotations and provides a principled interface between cellular heterogeneity and patient-level prediction.

Despite these strengths, several limitations remain. First, scCap performs clustering and phenotype prediction as sequential stages rather than within a fully end-to-end optimization framework. Jointly learning cluster assignments and phenotype prediction may further improve efficiency and enhance predictive consistency.

Second, the current knowledge augmentation strategy relies solely on representations derived from scGPT. Future work could incorporate complementary biological priors, including alternative single-cell foundation models or structured knowledge such as pathway relationships, ligand–receptor interactions, and regulatory networks, to further improve biological coherence and robustness.

Third, although scCap demonstrated consistent performance across three independent datasets, evaluation was conducted within each dataset using cross-validation. Assessing cross-dataset generalization, by training on one cohort and evaluating on external cohorts under potential distribution shifts, would provide a more stringent assessment of robustness and practical applicability.

In summary, scCap presents an annotation-free approach to phenotype prediction from scRNA-seq data by grounding predictive modeling in biologically structured, cluster-defined subpopulations. The results indicate that knowledge-augmented clustering serves as a principled intermediate representation linking single-cell heterogeneity to patient-level phenotypes.

**Key Points**

- scCap enables phenotype prediction without using predefined annotations during training by constructing knowledge-guided clusters directly from single-cell RNA-seq data, bypassing the need for subjective manual labeling.
- The framework employs a two-step knowledge-augmented clustering strategy, refining initial expression-based clusters using generalized representations from self-supervised single-cell foundation models as prior biological knowledge.
- By integrating these clusters into a hierarchical multiple instance learning framework with dual-level attention, scCap effectively links fine-grained cellular heterogeneity to patient-level phenotypes.
- scCap achieves robust predictive performance while enabling interpretable identification of disease-associated cell populations.

## Supplementary Material

0629_firstlook_supplementary_bbag395

## Data Availability

The COVID dataset is available at https://singlecell.broadinstitute.org/single_cell/study/SCP1289. The Cardio dataset is available at https://singlecell.broadinstitute.org/single_cell/study/SCP1303. The kidney dataset is available at https://cellxgene.cziscience.com/collections/0f528c8a-a25c-4840-8fa3-d156fa11086f. The source code is available at https://github.com/mjuailab/scCap.
