# Supplementary material for: Annotation-free phenotype prediction using knowledge-augmented clustering from single-cell RNA sequencing data
Source: Brief Bioinform. 2026 Jul 20;27(4):bbag395. doi: 10.1093/bib/bbag395 (PMC13384651; doi:10.1093/bib/bbag395)
Supplement: 0629_firstlook_supplementary_bbag395 [file 0629_firstlook_supplementary_bbag395.pdf]

Annotation-free phenotype prediction using knowledge-augmented clustering from single-cell RNA sequencing data: Supplementary Materials

Janghyun Noh, Yoobin Shin, Min Kim and Minsik Oh

1 Hyperparameter Settings and Optimization Strategy

To ensure a fair comparison, all models were trained and evaluated on identically pre-split datasets, following the same data partitioning across experiments. We employed a 5×5 nested cross-validation framework consisting of 5 outer folds and 5 inner folds, repeated five times to ensure stability of results. In each outer loop, one fold was held out as the test set, while the remaining folds were used for inner cross-validation. Within the inner folds, hyperparameters were optimized using Optuna, and the configuration yielding the highest mean AUROC across inner validation loops was selected as the optimal setting. Each model was then retrained on the entire outer training set with this configuration, and its performance was evaluated on the held-out outer test fold. This nested procedure was repeated five times with different random seeds, and the final performance was reported as the mean AUROC averaged across all outer folds and repetitions.

The hyperparameter settings explored for each model are summarized below [1-4].

- CloudPred:
  - Learning rate: {1e-4, 1e-3, 1e-2}
  - Number of epochs: {100}
  - Number of centers: {2, 8, 16}
- ProtoCell4P:
  - Learning rate: {1e-4, 1e-3, 1e-2}
  - Number of epochs: {50, 75, 100}
  - Hidden dimension: {8, 16, 32}
  - Latent dimension: {32, 64, 128}
  - Number of prototypes: {8, 16, 32}
  - Pretraining epochs: {75}
- ScRAT:
  - Learning rate: {1e-4, 1e-3, 1e-2}
  - Number of epochs: {100}
  - Number of attention heads: {1, 2, 4}
  - Dropout rate: {0.0, 0.3, 0.5, 0.7}
  - Weight decay: {1e-4, 1e-3, 1e-2}
  - Data augmentation: {True, False}
  - Embedding dimension: {8, 32, 64}
  - Number of augmented samples: {100}
  - PCA: {False}
- HA:
  - Number of epochs: {100, 500, 1000}
  - Dropout rate: {0.0, 0.3, 0.5, 0.7}
  - Weight decay: {1e-4, 1e-3, 1e-2}
  - Hidden dimensions: {32, 64, 128}
  - Number of linear input layers: {1, 2}

- Learning rate: {5e-3, 1e-3}

- scCap (Ours):
  - Number of epochs: {100, 500, 1000}
  - Dropout rate: {0.0, 0.3, 0.5, 0.7}
  - Weight decay: {1e-4, 1e-3, 1e-2, 1e-1}
  - Hidden dimensions: {32, 64, 128}
  - Number of linear input layers: {1, 2}
  - Learning rate: {5e-3, 1e-3}

2 Additional Performance Evaluation Results

To provide a more comprehensive evaluation of predictive performance, we report additional metrics including accuracy, precision, recall, and F1 score across all datasets and annotation settings. As shown in Table S1, scCap demonstrates consistently strong performance across these complementary metrics. Although certain annotation-based models achieve higher values for specific metrics in individual datasets, scCap maintains stable and competitive performance overall. These findings indicate that the proposed knowledge-augmented clustering framework achieves reliable predictive performance under multiple evaluation criteria.

In addition, we perform statistical significance tests using a paired t-test on results obtained across five pre-defined cross-validation splits. As summarized in Table S2, scCap consistently achieves higher performance than baseline methods across datasets and evaluation metrics, rather than only in selectively favorable cases. The results show statistically significant improvements over most baselines in the majority of settings, further supporting the effectiveness and robustness of the proposed approach.

Table S1. Comparison of additional performance metrics.

Metric	Method	COVID	Cardio	Kidney
Accuracy	CloudPred	0.71 ± 0.01	0.65 ± 0.04	0.67 ± 0.07
	ProtoCell4P	0.75 ± 0.08	0.71 ± 0.03	0.70 ± 0.05
	ScRAT	0.80 ± 0.10	0.79 ± 0.16	0.76 ± 0.12
	HA	0.80 ± 0.04	0.67 ± 0.05	0.81 ± 0.03
	scCap	**0.87 ± 0.04**	**0.85 ± 0.05**	**0.87 ± 0.04**
Precision	CloudPred	0.38 ± 0.05	0.51 ± 0.05	0.47 ± 0.10
	ProtoCell4P	0.72 ± 0.08	0.71 ± 0.03	0.67 ± 0.06
	ScRAT	0.86 ± 0.11	0.71 ± 0.26	0.62 ± 0.21
	HA	0.74 ± 0.09	0.63 ± 0.06	0.74 ± 0.04
	scCap	**0.88 ± 0.04**	**0.85 ± 0.06**	**0.83 ± 0.07**
Recall	CloudPred	0.52 ± 0.02	0.60 ± 0.03	0.55 ± 0.08
	ProtoCell4P	0.71 ± 0.08	0.71 ± 0.02	0.67 ± 0.05
	ScRAT	**0.88 ± 0.13**	0.75 ± 0.17	0.66 ± 0.15
	HA	0.72 ± 0.05	0.67 ± 0.04	0.71 ± 0.03
	scCap	0.81 ± 0.05	**0.84 ± 0.04**	**0.79 ± 0.05**
F1 Score	CloudPred	0.44 ± 0.03	0.53 ± 0.04	0.48 ± 0.09
	ProtoCell4P	0.70 ± 0.10	0.71 ± 0.03	0.66 ± 0.05
	ScRAT	**0.86 ± 0.07**	0.71 ± 0.22	0.62 ± 0.18
	HA	0.71 ± 0.07	0.62 ± 0.05	0.71 ± 0.03
	scCap	0.82 ± 0.05	**0.82 ± 0.06**	**0.79 ± 0.06**

Table S2. Statistical significance of scCap compared to baseline methods across datasets.

Metric	Dataset	CloudPred	ProtoCell4P	ScRAT	HA
	COVID	<0.05	<0.01	0.156	0.057
AUROC	Cardio	<0.01	<0.01	0.195	<0.01
	Kidney	<0.05	<0.01	0.068	<0.01
	COVID	<0.05	<0.01	0.072	<0.05
AUPRC	Cardio	<0.01	<0.01	0.214	<0.01
	Kidney	0.082	<0.01	<0.05	0.080
	COVID	<0.01	<0.01	0.083	<0.01
Accuracy	Cardio	<0.01	<0.01	0.439	<0.01
	Kidney	<0.01	<0.01	<0.05	<0.01
	COVID	<0.01	<0.01	0.455	<0.01
Precision	Cardio	<0.01	<0.01	0.099	<0.01
	Kidney	<0.01	<0.01	<0.01	<0.01
	COVID	<0.01	<0.05	<0.01	<0.05
Recall	Cardio	<0.01	<0.01	0.246	<0.05
	Kidney	<0.01	<0.01	<0.05	<0.01
	COVID	<0.01	<0.01	<0.05	<0.01
F1	Cardio	<0.01	<0.05	0.197	<0.01
	Kidney	<0.01	<0.01	<0.05	<0.01

3 Performance Comparison Across Annotation Conditions

To assess the robustness of scCap with respect to annotation sources, we compared three settings: (i) manual annotations, (ii) SingleR-derived annotations, and (iii) the proposed annotation-free refined clustering. All experiments used identical model architecture, training protocol, and data splits only the annotation source used to define clusters differed.

Across datasets and evaluation metrics (AUROC, AUPRC, Accuracy, Precision, Recall, and F1 score), the annotation-free configuration achieved competitive or superior performance (Table S3). These findings indicate that clusters obtained through knowledge-augmented refinement provide representations that are at least as informative as predefined annotations for phenotype prediction.

In addition, Table S4 provides a focused comparison of annotation-based methods under manual and SingleR annotations. The results show observable performance differences across annotation sources, suggesting that annotation quality can influence downstream phenotype prediction performance.

Table S3. Performance Comparison of scCap Across Annotation Conditions.

	AUROC			AUPRC			Accuracy		
Dataset	Manual	SingleR	Free	Manual	SingleR	Free	Manual	SingleR	Free
COVID	0.87 ± 0.02	0.89 ± 0.03	$\mathbf{0.93 \pm 0.03}$	0.94 ± 0.01	0.96 ± 0.02	$\mathbf{0.97 \pm 0.01}$	0.84 ± 0.02	0.82 ± 0.03	$\mathbf{0.87 \pm 0.04}$
Cardio	0.93 ± 0.01	0.94 ± 0.04	$\mathbf{0.97 \pm 0.01}$	0.90 ± 0.02	0.92 ± 0.05	$\mathbf{0.96 \pm 0.01}$	0.83 ± 0.03	0.80 ± 0.04	$\mathbf{0.85 \pm 0.05}$
Kidney	$\mathbf{0.94 \pm 0.02}$	0.92 ± 0.04	$\mathbf{0.94 \pm 0.02}$	0.89 ± 0.03	0.88 ± 0.05	$\mathbf{0.91 \pm 0.04}$	0.85 ± 0.03	0.85 ± 0.04	$\mathbf{0.87 \pm 0.04}$

	Precision			Recall			F1 Score		
Dataset	Manual	SingleR	Free	Manual	SingleR	Free	Manual	SingleR	Free
COVID	0.80 ± 0.05	0.80 ± 0.03	$\mathbf{0.88 \pm 0.04}$	0.77 ± 0.04	0.76 ± 0.04	$\mathbf{0.81 \pm 0.05}$	0.77 ± 0.04	0.75 ± 0.04	$\mathbf{0.82 \pm 0.05}$
Cardio	0.83 ± 0.04	0.76 ± 0.07	$\mathbf{0.85 \pm 0.06}$	0.82 ± 0.03	0.77 ± 0.05	$\mathbf{0.84 \pm 0.04}$	0.80 ± 0.04	0.74 ± 0.06	$\mathbf{0.82 \pm 0.06}$
Kidney	0.78 ± 0.07	0.79 ± 0.03	$\mathbf{0.83 \pm 0.07}$	0.76 ± 0.06	0.77 ± 0.04	$\mathbf{0.79 \pm 0.05}$	0.74 ± 0.06	0.76 ± 0.04	$\mathbf{0.79 \pm 0.06}$

Table S4. Sensitivity of Annotation-based Methods to Annotation Sources.

Method	Annotation	COVID	Cardio	Kidney
ProtoCell4P	Manual	0.80 ± 0.07	0.88 ± 0.02	0.83 ± 0.05
	SingleR	0.81 ± 0.07	0.87 ± 0.02	0.83 ± 0.05
ScRAT	Manual	0.86 ± 0.21	0.93 ± 0.10	0.88 ± 0.10
	SingleR	0.86 ± 0.21	0.93 ± 0.10	0.85 ± 0.02
HA	Manual	0.89 ± 0.05	0.87 ± 0.02	0.92 ± 0.01
	SingleR	0.87 ± 0.05	0.90 ± 0.04	0.90 ± 0.01

4 Biological Interpretation of Significant Subpopulations

To further assess the biological interpretability of scCap, we examined the biological relevance of the significant subpopulations identified in each dataset. For both datasets, we first quantified cluster-level importance scores to evaluate each cluster's contribution to phenotype prediction, where positive and negative scores indicate stronger associations with disease and normal samples, respectively. Clusters with the five largest absolute importance scores were defined as significant subpopulations.

Among these, we focused on the disease-associated cluster with the highest positive importance score in each dataset as a representative case for in-depth biological interpretation. For this top disease-associated subpopulation, we inferred its potential cell type and underlying cellular program based on overexpressed canonical marker genes, and then performed differential expression analysis between clinical groups restricted to this cluster to assess whether its transcriptional profile captures disease-relevant molecular signals. The following subsections present these analyses for the Cardio datset [5] with analogous results for the Kidney dataset [10] provided in the Supplementary Information.

4.1 Cardio dataset

In the Cardio dataset, we first quantified cluster-level importance scores to assess each cluster's contribution to phenotype prediction. The five clusters with the largest absolute importance scores were defined as significant subpopulations (Figure S1). Their locations in the learned UMAP space are shown in Figure S2, and their potential cell types were inferred from the expression of canonical marker genes overexpressed within each cluster (Figure S3).

Among these significant subpopulations, cluster 6 showed the highest positive importance score, indicating a strong association with disease phenotypes. Based on high expression of epicardial marker genes such as *ALDH1A2, WT1*, and *BNC1*, we inferred that this cluster corresponds to epicardial-derived cells rather than cardiomyocytes or endothelial cells [6]. Epicardial cells normally form a protective layer covering the heart surface, but under pathological conditions they can undergo an epicardial-to-mesenchymal transition and give rise to fibroblast-like cells that secrete extracellular matrix proteins and promote cardiac fibrosis [7]. These observations suggest that scCap has identified an epicardial-derived subpopulation with a high propensity for fibrosis that is closely linked to disease.

To further examine whether this epicardial subpopulation captures molecular signals relevant to cardiac pathology, we performed differential expression analysis between Normal, HCM, and DCM samples within cluster 6. We observed disease-associated upregulation of complement component *C3* and the metal transporter *SLC39A8*, both of which have been implicated in adverse cardiovascular outcomes. *C3* is a central effector of the complement cascade, and higher serum *C3* concentrations have been associated with pre-existing severe coronary artery disease as well as an increased risk of new vascular events in women [8]. Likewise, a common missense variant in *SLC39A8* has been linked to altered circulating lipid levels and an increased risk of coronary artery disease in large-scale genetic association studies [9]. Together, these findings indicate that cluster 6 represents an epicardial-derived, fibrosis-prone subpopulation that is not only biologically interpretable at the cell-type level, but also enriched for disease-relevant inflammatory and metabolic signals (Figure S4).

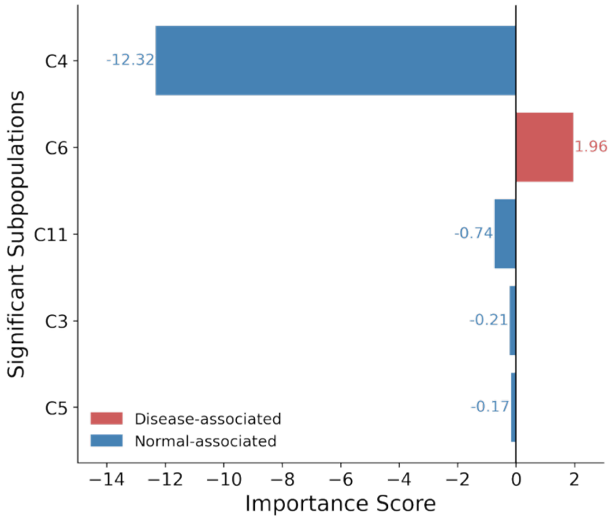

Figure S1. Importance score of identified significant subpopulations in the Cardio dataset.

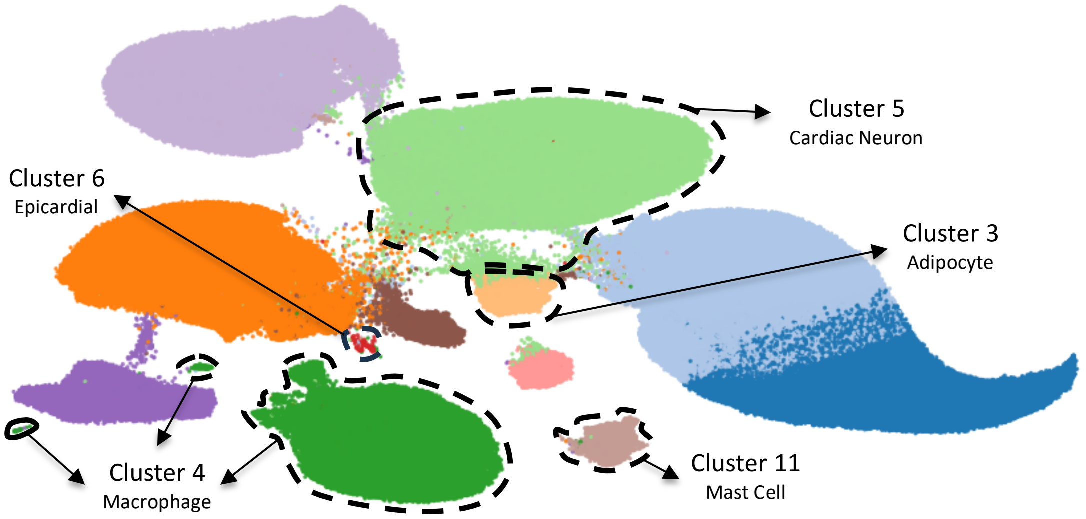

Figure S2. UMAP visualization of significant subpopulations in the Cardio dataset.

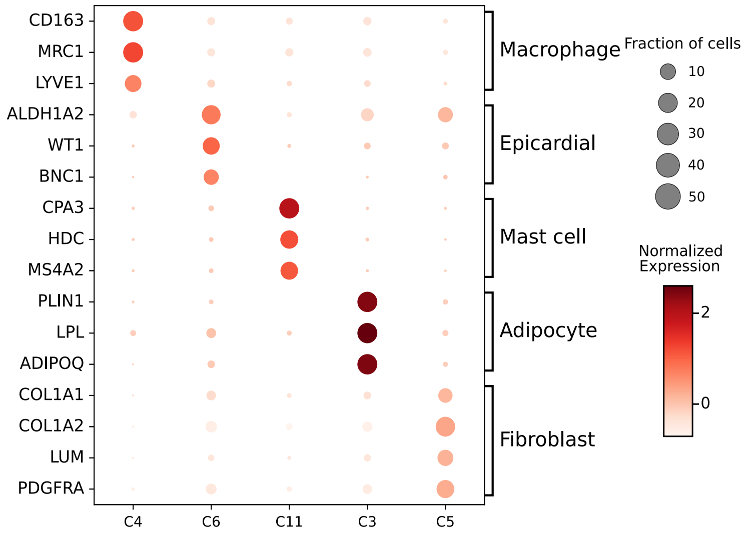

Figure S3. Potential cell type characterization of significant subpopulations identified in the Cardio dataset.

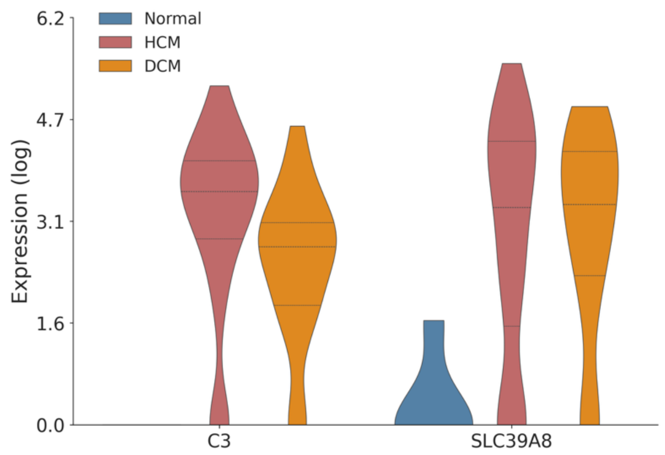

Figure S4. Differential expression analysis of top disease-associated subpopulation in the Cardio dataset.

4.2 Kidney dataset

Following the same analytical procedure, we computed importance scores for all clusters in the Kidney dataset. Clusters with the five largest absolute scores were defined as significant subpopulations (Figure S5), and their locations in the learned UMAP space are shown in Figure S6.

To infer the potential cell types represented by the significant subpopulations in the Kidney dataset, we examined the expression of canonical kidney marker genes (Figure S7). Among these subpopulations, we focused on cluster 4, which showed the highest positive importance score among disease-associated clusters. Cluster 4 displayed marked overexpression of the inflammatory monocyte markers *S100A8* and *S100A9*, consistent with a monocyte population enriched for tissue-infiltrating, pro-inflammatory cells that have been described in human kidney single-cell atlases [11]. This expression pattern indicates that scCap assigns the strongest disease importance to a distinct inflammatory monocyte subpopulation, suggesting that activation or expansion of this compartment is a key immune feature of the diseased kidney.

To further examine whether this inflammatory monocyte subpopulation captures kidney disease–relevant molecular signals, we performed differential expression analysis between Normal, CKD, and AKF samples restricted to cluster 4. This analysis revealed strong disease-associated upregulation of *FKBP5* and *CXCR4* in CKD and AKF compared with Normal (Figure S8). FKBP5 encodes FKBP51, an Hsp90-associated co-chaperone that modulates NF-κB signaling and glucocorticoid receptor sensitivity, and experimental work has shown that targeting FKBP5 with microRNA-23a-3p ameliorates sepsis-induced acute kidney injury by dampening NF-κB–mediated inflammatory response [12]. *CXCR4* encodes the canonical receptor for the chemokine *CXCL12*, and the CXCL12–CXCR4 axis has been implicated in leukocyte recruitment, persistent inflammation, and fibrotic remodeling across multiple forms of kidney injury, including AKI and CKD [13]. The coordinated upregulation of *FKBP5* and *CXCR4* within cluster 4 therefore suggests that scCap has identified a kidney monocyte subpopulation that is primed for chemokine-driven recruitment and NF-κB–dependent inflammatory activation, providing a mechanistic link between this subpopulation and the progression of CKD and AKF.

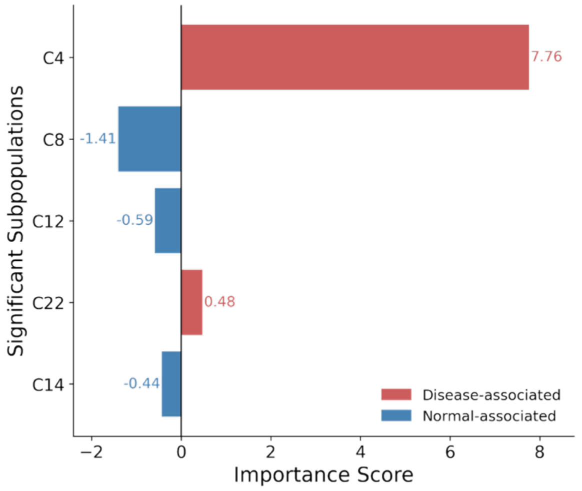

Figure S5. Importance score of identified significant subpopulations in the Kidney dataset.

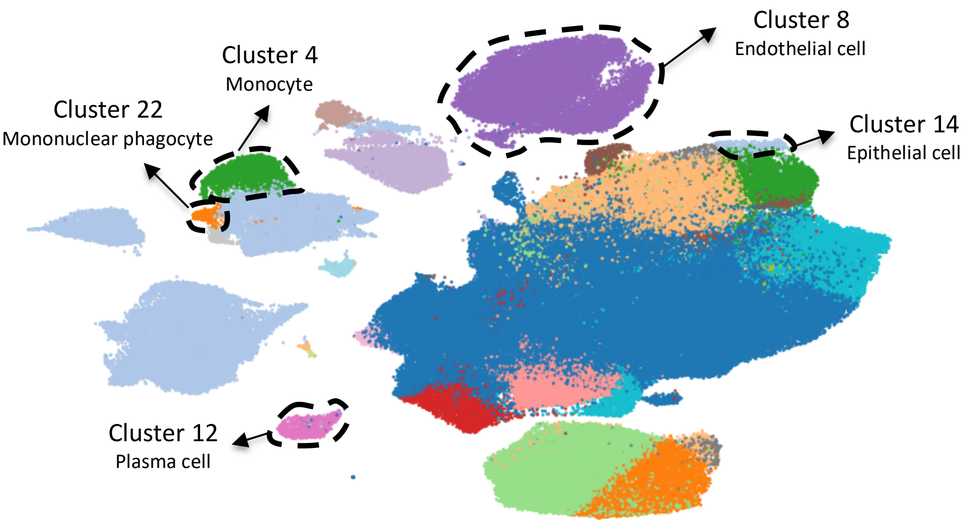

Figure S6. UMAP visualization of significant subpopulations in the Kidney dataset.

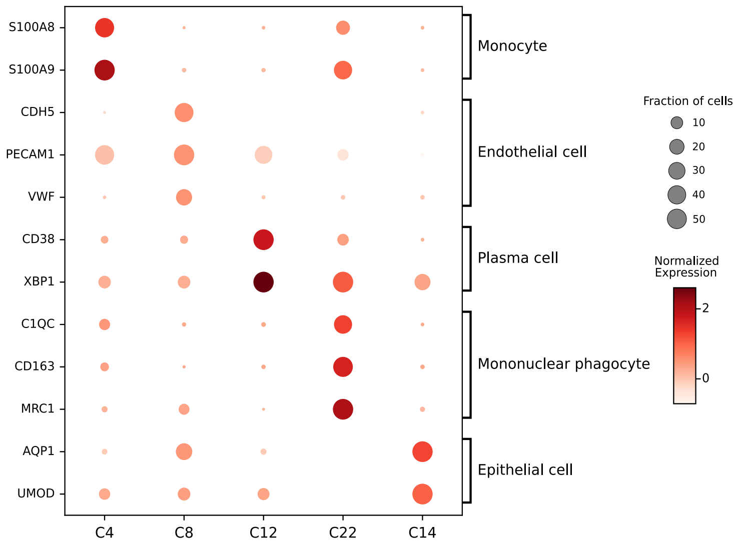

Figure S7. Potential cell type characterization of significant subpopulations identified in the Kidney dataset.

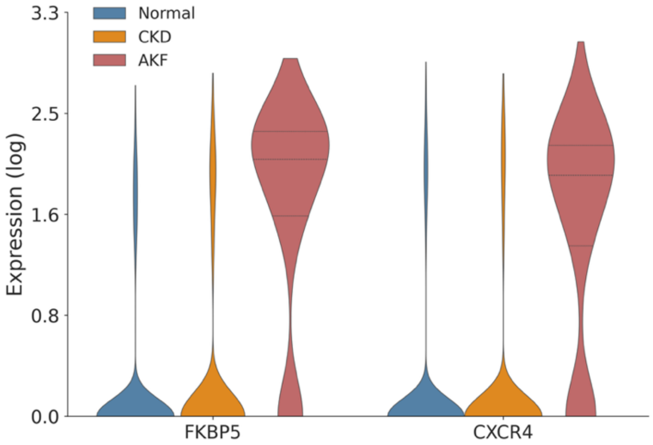

Figure S8. Differential expression analysis of top disease-associated subpopulation in the Kidney dataset.

5 Interpretability Comparison with Baseline Models

To assess the biological relevance of the subpopulations identified by scCap, we qualitatively compared its interpretation with representative baseline models on the COVID dataset.

ProtoCell4P [2] quantifies cell-type importance using prototype-based contribution scores derived from predefined annotations. In their analysis, mast cells (3.25), secretory cells (2.60), and goblet cells (2.20) were assigned the highest contribution scores. Meanwhile, ciliated epithelial cells received a comparatively lower score (0.83), despite prior studies reporting that ciliated cells exhibit substantial transcriptional changes during SARS-CoV-2 infection [14].

In contrast, the hierarchical attention (HA) framework [4] estimates importance through dual-level attention mechanisms over cells and cell types. HA successfully highlights epithelial populations as important contributors; however, importance is aggregated at the predefined cell-type level, without explicitly resolving heterogeneity within the same lineage.

In comparison, scCap does not rely on predefined cell-type categories but instead learns from knowledge-augmented clustering to estimate importance. As a result, it structurally captures the basal–developing–ciliated trajectory associated with ciliated regeneration and identifies multiple lineage-related subpopulations as highly influential clusters.

Furthermore, as illustrated in Figures S9 and S10, scCap does not treat the ciliated lineage as a single homogeneous group. Instead, it decomposes transcriptionally distinct states within the lineage and assigns differential importance scores to each subpopulation. This indicates that the model reflects not only lineage structure but also state-specific heterogeneity within the same lineage during phenotype prediction.

Taken together, these results demonstrate that scCap moves beyond cell-type-level importance estimation by integrating lineage organization and state-specific variation, thereby enabling a more precise and structurally coherent identification of disease-associated cellular programs.

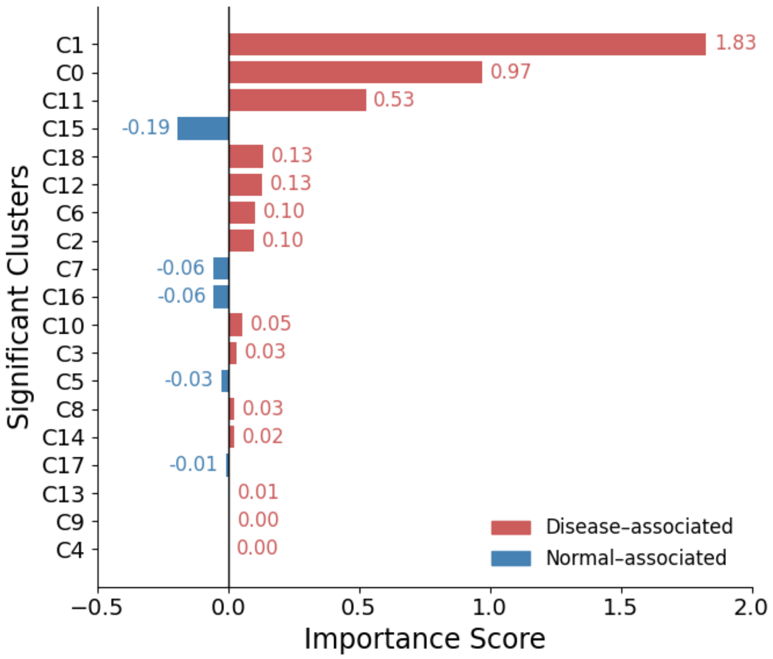

Figure S9. Importance score of identified significant subpopulations in the COVID dataset.

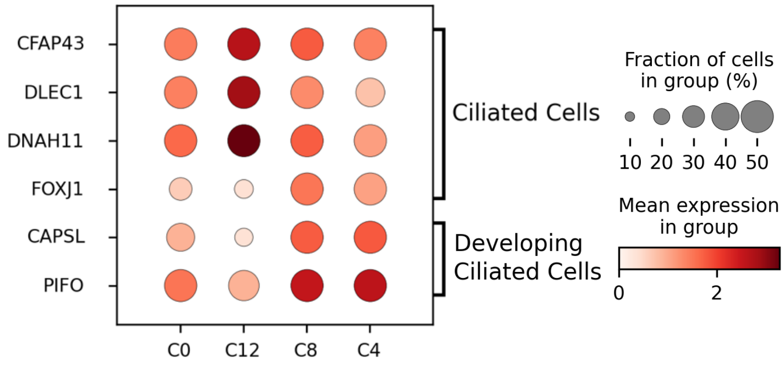

Figure S10. State-Specific Characterization of Ciliated Subpopulations Identified in the COVID dataset.

6 Sensitivity and Structural Robustness Analysis of Clustering

To evaluate the robustness of scCap with respect to clustering-related hyperparameters, we conducted systematic sensitivity analyses on: (i) the resolution parameter that determines the number of initial clusters, (ii) the dispersion threshold controlling the cluster splitting process, and (iii) the merge ratio controlling the refinement step in knowledge-augmented clustering.

6.1 Resolution Sensitivity

The resolution parameter directly controls the granularity of the initial clustering structure. We varied the resolution across a wide range of values for each dataset, resulting in substantially different numbers of clusters.

Despite these variations in cluster granularity, scCap maintained stable predictive performance across AUROC, AUPRC, Accuracy, Precision, Recall, and F1 score.

Across the COVID, Cardio, and Kidney datasets, performance fluctuations were minor and no consistent degradation was observed at either coarse or fine resolutions.

These findings indicate that scCap does not rely on delicate tuning of the initial cluster size and remains robust under varying clustering granularities.

Table S5. Sensitivity of scCap to the Initial Clustering Resolution.

Dataset	Resolution	Clusters	AUROC	AUPRC	Accuracy	Precision	Recall	F1
COVID	0.5	14	0.95 ± 0.02	**0.98 ± 0.01**	0.88 ± 0.03	0.88 ± 0.04	0.84 ± 0.04	0.84 ± 0.04
	1	16	0.93 ± 0.03	0.97 ± 0.01	0.87 ± 0.04	0.88 ± 0.04	0.81 ± 0.05	0.82 ± 0.05
	1.5	18	0.94 ± 0.05	0.97 ± 0.03	**0.90 ± 0.04**	**0.90 ± 0.06**	**0.85 ± 0.06**	**0.85 ± 0.06**
	2	19	**0.96 ± 0.03**	**0.98 ± 0.01**	0.88 ± 0.02	0.89 ± 0.03	0.82 ± 0.03	0.83 ± 0.04
Cardio	0.5	15	0.96 ± 0.03	0.94 ± 0.04	0.84 ± 0.05	0.82 ± 0.09	0.83 ± 0.06	0.80 ± 0.08
	1	12	0.97 ± 0.00	0.96 ± 0.01	**0.85 ± 0.05**	**0.85 ± 0.06**	**0.84 ± 0.04**	**0.82 ± 0.06**
	1.5	13	**0.98 ± 0.02**	**0.97 ± 0.03**	0.83 ± 0.05	0.82 ± 0.09	0.83 ± 0.05	0.80 ± 0.08
	2	15	0.96 ± 0.03	0.95 ± 0.04	0.84 ± 0.02	0.83 ± 0.03	**0.84 ± 0.02**	0.81 ± 0.02
Kidney	0.5	23	0.93 ± 0.02	0.90 ± 0.03	**0.87 ± 0.02**	**0.85 ± 0.05**	**0.81 ± 0.03**	**0.81 ± 0.03**
	1	24	**0.94 ± 0.02**	**0.91 ± 0.04**	**0.87 ± 0.04**	0.83 ± 0.07	0.79 ± 0.05	0.79 ± 0.06
	1.5	34	0.93 ± 0.01	0.89 ± 0.01	**0.87 ± 0.03**	0.81 ± 0.06	0.78 ± 0.06	0.77 ± 0.06
	2	29	0.91 ± 0.01	0.88 ± 0.03	0.83 ± 0.04	0.78 ± 0.08	0.76 ± 0.05	0.74 ± 0.07

6.2 Splitting Threshold Sensitivity

To evaluate the robustness of the cluster splitting criterion, we analyze the effect of varying the dispersion threshold across a range of values (0.3, 0.5, and 0.7).

As shown in Table S6, the number of clusters after the splitting step varies depending on the threshold, where lower values lead to more aggressive splitting and higher values result in more conservative behavior. This effect is particularly evident in the Cardio dataset, where the number of split clusters decreases substantially as the threshold increases.

However, despite these differences in intermediate cluster granularity, the final number of refined clusters remains largely stable across different threshold settings. This indicates that the subsequent merging process effectively consolidates similar clusters and mitigates the impact of over- or under-splitting.

We note that in the Kidney dataset, slight variations in the number of refined clusters are observed across thresholds. Nevertheless, clustering quality metrics show that a threshold of 0.5 consistently achieves the best overall performance, suggesting a more favorable balance between cluster granularity and structural coherence.

Overall, these results demonstrate that the clustering framework is robust to the choice of dispersion threshold. Based on this observation, we use a threshold of 0.5 in all experiments as a balanced setting that avoids excessive fragmentation while preserving meaningful cluster structure.

Table S6. Sensitivity of scCap to the Splitting Threshold.

		Cluster Count			Clustering Metrics				
Dataset	Dispersion	Init	Split	Refined	Silhouette	DB	AMI	Homogeneity	Completeness
COVID	0.3	21	42	19	0.0911	1.7205	0.459	0.4387	0.4841
	0.5	21	42	19	0.0911	1.7205	0.459	0.4387	0.4841
	0.7	21	42	19	0.0911	1.7205	0.459	0.4387	0.4841
Cardio	0.3	21	42	13	0.3639	1.1052	0.9166	0.9414	0.8931
	0.5	21	39	13	0.3639	1.1052	0.9166	0.9414	0.8931
	0.7	21	27	13	0.3639	1.1052	0.9166	0.9414	0.8931
Kidney	0.3	32	64	20	0.0720	1.5605	0.6194	0.5378	0.7309
	0.5	32	64	20	**0.0720**	**1.5605**	**0.6194**	**0.5378**	**0.7309**
	0.7	32	62	21	0.0248	1.5808	0.6065	0.5356	0.6995

6.3 Merge Ratio Sensitivity

We further examined the effect of the merge ratio used during cluster refinement. This parameter determines how aggressively similar clusters are merged after the initial split step. We compared the initial clustering, split-only

configuration, and multiple merging values (ρ).

Across datasets, refinement consistently improved predictive performance relative to the initial clustering. Importantly, varying the merge ratio did not introduce instability, as performance metrics remained comparable across different settings.

However, different values of ρ result in varying numbers of final clusters. In particular, larger values of ρ tend to produce more clusters, while smaller values lead to more aggressive merging and fewer clusters.

Given that predictive performance remains stable, we select the merge ratio based on structural interpretability. Specifically, we choose $\rho = 2.0$, as it yields a number of clusters that is most consistent with the number of manually annotated cell types in each dataset (e.g., COVID: 18, Cardio: 13, Kidney: 25).

Overall, these results indicate that scCap is robust to the choice of merge ratio, while $\rho = 2.0$ provides a balanced setting that preserves biological granularity without introducing unnecessary fragmentation.

Table S7. Sensitivity of scCap to the Cluster Refinement Merge Ratio.

Dataset	Merge	Clusters	AUROC	AUPRC	Accuracy	Precision	Recall	F1
	Initial	22	0.89 ± 0.03	0.96 ± 0.01	0.84 ± 0.03	0.81 ± 0.06	0.77 ± 0.05	0.76 ± 0.05
	Split	44	0.91 ± 0.03	0.96 ± 0.02	0.84 ± 0.04	0.86 ± 0.03	0.80 ± 0.04	0.80 ± 0.04
COVID	2	19	0.93 ± 0.03	0.97 ± 0.01	**0.87 ± 0.04**	**0.88 ± 0.04**	0.81 ± 0.05	**0.82 ± 0.05**
	2.5	21	**0.95 ± 0.02**	**0.98 ± 0.01**	0.86 ± 0.03	0.86 ± 0.04	0.81 ± 0.03	0.81 ± 0.03
	3	33	0.94 ± 0.05	0.97 ± 0.02	0.86 ± 0.02	0.86 ± 0.04	**0.83 ± 0.03**	**0.82 ± 0.03**
	Initial	20	0.96 ± 0.02	0.95 ± 0.02	0.87 ± 0.02	0.87 ± 0.03	0.86 ± 0.03	0.84 ± 0.04
	Split	35	0.96 ± 0.02	0.94 ± 0.02	**0.94 ± 0.02**	0.88 ± 0.04	0.86 ± 0.03	0.84 ± 0.03
Cardio	2	12	**0.97 ± 0.00**	**0.96 ± 0.01**	0.85 ± 0.05	0.85 ± 0.06	0.84 ± 0.04	0.82 ± 0.06
	2.5	20	**0.97 ± 0.01**	**0.96 ± 0.02**	0.85 ± 0.05	0.88 ± 0.05	0.86 ± 0.04	0.83 ± 0.06
	3	21	**0.97 ± 0.02**	**0.96 ± 0.03**	0.88 ± 0.05	**0.90 ± 0.05**	**0.88 ± 0.05**	**0.86 ± 0.06**
	Initial	34	0.92 ± 0.03	0.88 ± 0.04	0.86 ± 0.02	0.82 ± 0.07	0.79 ± 0.03	0.78 ± 0.05
	Split	68	0.94 ± 0.01	**0.92 ± 0.01**	0.83 ± 0.02	0.75 ± 0.09	0.75 ± 0.05	0.73 ± 0.06
Kidney	2	24	0.94 ± 0.02	0.91 ± 0.04	0.87 ± 0.04	0.83 ± 0.07	0.79 ± 0.05	0.79 ± 0.06
	2.5	42	0.94 ± 0.01	0.91 ± 0.02	0.86 ± 0.03	0.82 ± 0.07	0.79 ± 0.04	0.78 ± 0.05
	3	57	**0.95 ± 0.01**	**0.92 ± 0.02**	**0.89 ± 0.02**	**0.87 ± 0.06**	**0.84 ± 0.03**	**0.84 ± 0.03**

7 Analysis of Rare Subpopulation Identification

Single-cell RNA sequencing is particularly useful for identifying rare cell populations, which are often difficult to detect due to their low abundance. To evaluate whether scCap can capture such rare subpopulations, we perform additional analysis across all datasets using manual cell type annotations (Figures S11–S13).

We analyze how rare cell types are distributed across the refined clusters, focusing on whether they are dispersed or concentrated. As shown in Figures S11-S13, several rare cell populations tend to be concentrated within specific clusters rather than being uniformly dispersed. In many cases, a substantial proportion of these cells is assigned to a single cluster.

Quantitatively, we find that a notable fraction of rare cells is assigned to their dominant cluster. For example, as shown in Figure S13, in the Kidney dataset, plasma cells, which account for only 0.47% of the total cells, exhibit strong concentration, with 97.3% assigned to a single cluster (C12). This cluster is also identified as important in the model's prediction. These observations demonstrate that scCap can effectively identify and preserve rare cell populations, while also capturing their contribution to downstream phenotype prediction, even for extremely rare cell types.

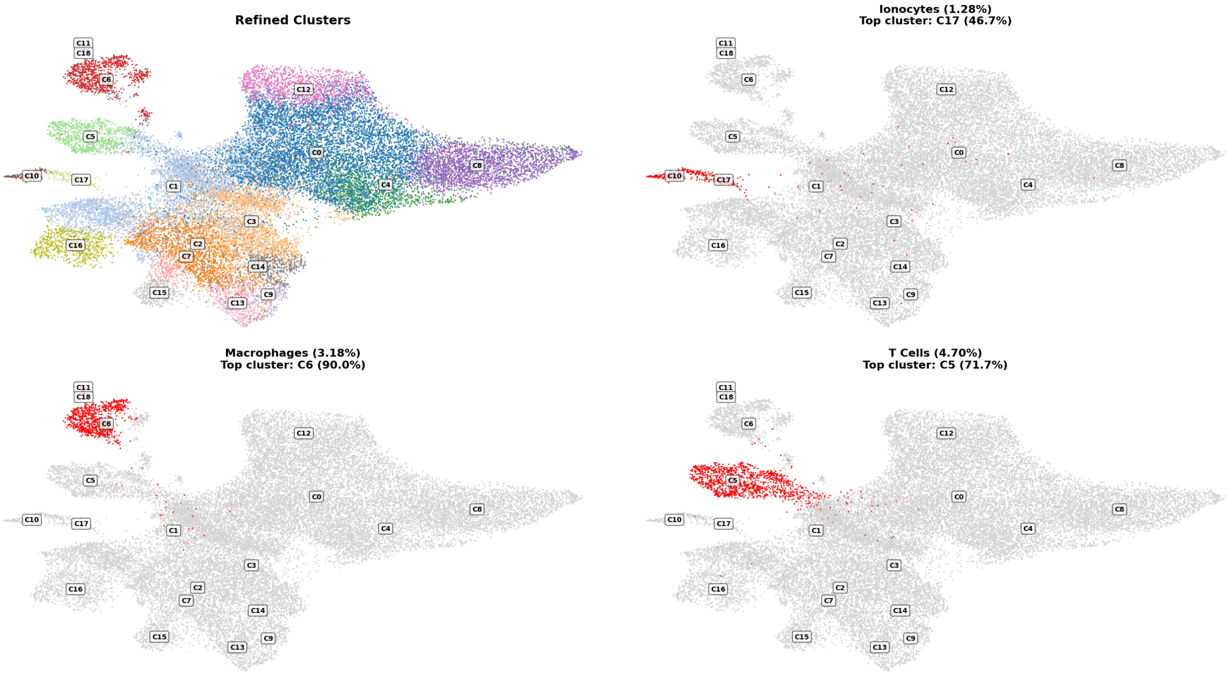

Figure S11. Rare cell distribution across refined clusters in the COVID dataset.

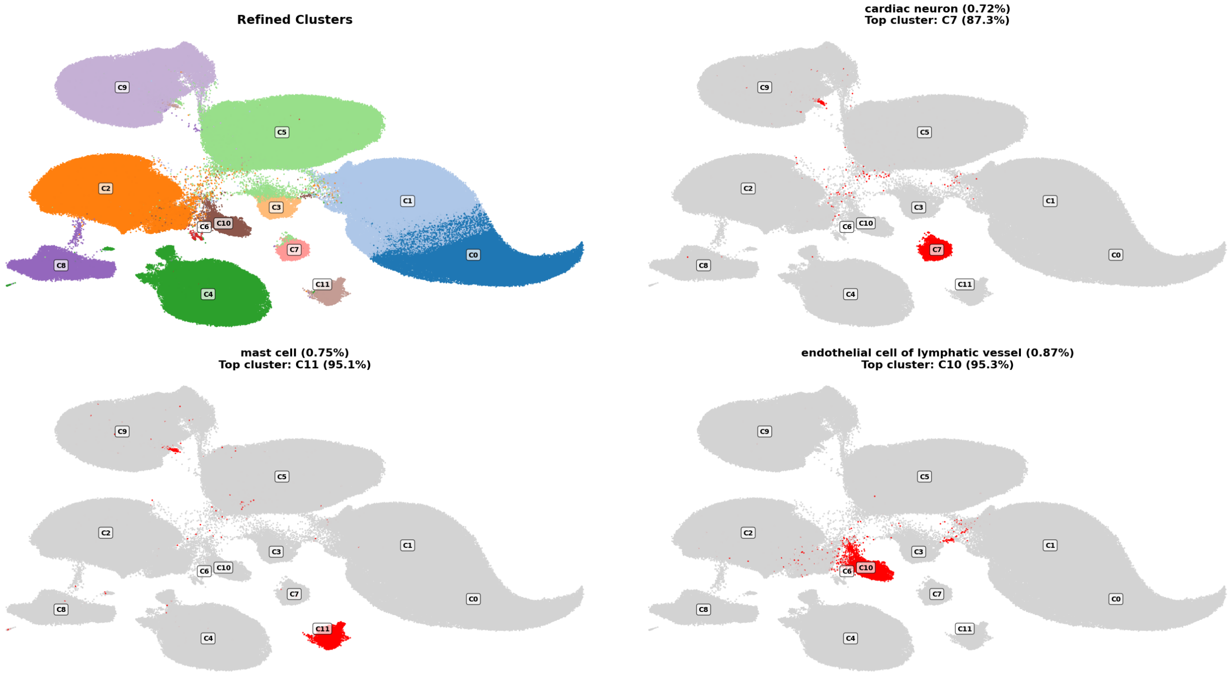

Figure S12. Rare cell distribution across refined clusters in the Cardio dataset.

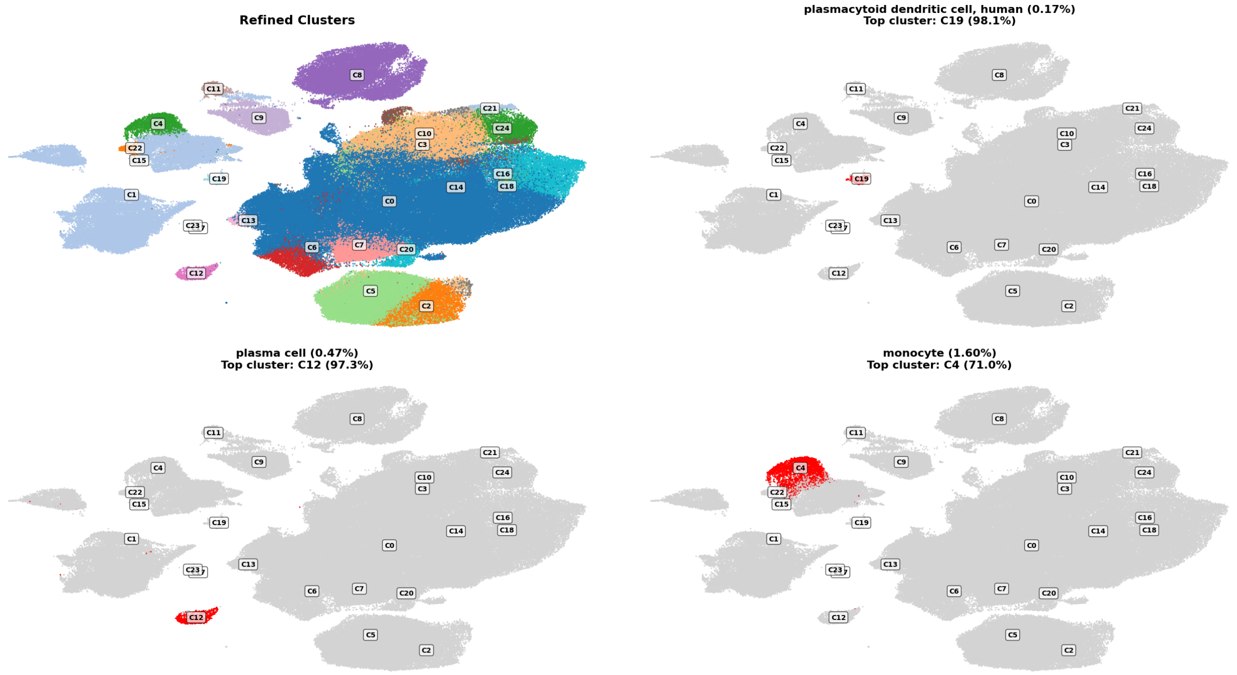

Figure S13. Rare cell distribution across refined clusters in the Kidney dataset.

8 Computational Efficiency and Runtime Analysis

To further analyze the computational efficiency of scCap, we report runtime comparisons with baseline methods across datasets, together with the experimental hardware configuration and additional preprocessing costs.

All experiments were conducted on a server equipped with 4× NVIDIA RTX 4090 GPUs, where each experiment utilized a single GPU along with standard CPU resources. This setup reflects a commonly accessible research environment and ensures reproducibility without requiring specialized hardware.

We report the average runtime over five repeated runs, excluding preprocessing procedures, to provide a fair comparison of the core phenotype prediction models. As summarized in Table S8, scCap requires moderately longer runtime than lightweight baseline methods such as ScRAT and CloudPred, mainly due to its hierarchical dual-attention architecture. Nevertheless, scCap remains substantially more efficient than computationally intensive approaches such as ProtoCell4P.

In addition, scCap includes an additional clustering refinement procedure prior to model training, requiring approximately 5 minutes for COVID, 50 minutes for Cardio, and 20 minutes for Kidney. Since this preprocessing step is performed separately before training, it is not included in the reported runtime measurements.

To further characterize the computational requirements of scCap, we measured the peak GPU memory and RAM usage during model execution. The measured resource usage and corresponding hardware recommendations are summarized in Table S9. Resource requirements generally increased with dataset size, reflecting the additional memory and computation required to process larger numbers of cells within the hierarchical MIL framework.

Overall, these results demonstrate that scCap achieves a practical trade-off between predictive performance and computational efficiency for real-world single-cell analysis applications.

Table S8. Average runtime comparison of scCap and baseline methods across datasets.

Dataset	CloudPred	ProtoCell4P	ScRAT	HA	scCap
COVID	39m	2h 28m	24m	1h 4m	1h 11m
Cardio	44m	40h 15m	38m	2h 7m	3h 52m
Kidney	1h 18m	20h 28m	44m	1h 9m	1h 38m

Table S9. Measured resource usage and recommended hardware requirements of scCap.

Dataset	Cells	Peak GPU	Peak RAM	Recommended GPU	Recommended RAM
COVID	26,947	1.73 GB	2.45 GB	$<$ 4 GB	$<$ 4 GB
Kidney	225,177	8.58 GB	16.64 GB	$<$ 16 GB	$<$ 32 GB
Cardio	592,689	20.33 GB	35.42 GB	$<$ 24 GB	$<$ 48 GB

9 Cluster Quality Analysis of the Refinement Procedure

To provide a more granular assessment of the proposed refinement procedure, we additionally evaluated intermediate clustering quality metrics across the initial, split, and refined clustering results. Specifically, we report the Silhouette score, Davies–Bouldin (DB) index, Adjusted Mutual Information (AMI), Homogeneity, and Completeness for each dataset, as summarized in Table S10.

Overall, the proposed split–merge strategy consistently improves clustering compactness and separation quality, as evidenced by higher Silhouette scores and lower DB indices across datasets. Refined clusters also exhibit consistently improved Completeness, suggesting that the proposed refinement procedure produces more globally coherent cluster structures.

In contrast, annotation-alignment metrics such as AMI and Homogeneity decrease after refinement in the COVID and Kidney datasets. This behavior indicates a trade-off between strict agreement with predefined annotations and refinement of the intrinsic cluster structure. Since the proposed framework refines clusters using scGPT embeddings that capture transcriptomic similarity patterns rather than directly optimizing annotation agreement, the refinement process may reorganize cells according to latent transcriptomic relationships that are not fully aligned with predefined annotation boundaries. Consequently, the refinement procedure appears to improve cluster compactness and structural coherence while not necessarily maximizing agreement with predefined annotations.

Table S10. Cluster quality analysis of the proposed refinement process.

Dataset	Cluster	Silhouette	DB	AMI	Homogeneity	Completeness
COVID	Initial	0.0164	3.5644	**0.5246**	0.6027	0.4664
	Split	0.0120	3.7992	0.4910	**0.6424**	0.4004
	Refined	**0.0911**	**1.7205**	0.4590	0.4387	**0.4841**
Cardio	Initial	0.1147	2.2684	0.8288	0.9695	0.7238
	Split	0.0611	2.9933	0.7486	**0.9703**	0.6094
	Refined	**0.3639**	**1.1052**	**0.9166**	0.9414	**0.8931**
Kidney	Initial	0.0248	2.6095	**0.7077**	0.8125	0.6271
	Split	0.0438	2.6381	0.6409	**0.8260**	0.5241
	Refined	**0.0612**	**1.5605**	0.6194	0.5378	**0.7309**

References

[1] He, Bryan, et al. "Cloudpred: Predicting patient phenotypes from single-cell rna-seq." PACIFIC SYMPOSIUM ON BIOCOMPUTING 2022. 2021.

[2] Xiong, Guangzhi, Stefan Bekiranov, and Aidong Zhang. "ProtoCell4P: an explainable prototype-based neural network for patient classification using single-cell RNA-seq." Bioinformatics 39.8 (2023): btad493.

[3] Mao, Yuzhen, et al. "Phenotype prediction from single-cell RNA-seq data using attention-based neural networks." Bioinformatics 40.2 (2024): btae067.

[4] Do, Chau, and Harri Lähdesmäki. "Incorporating hierarchical information into multiple instance learning for patient phenotype prediction with single-cell RNA-sequencing data." Bioinformatics 41.Supplement_1 (2025): i96-i104.

[5] Chaffin, Mark, et al. "Single-nucleus profiling of human dilated and hypertrophic cardiomyopathy." Nature 608.7921 (2022): 174-180.

[6] Meier, Anna B., et al. "Epicardioid single-cell genomics uncovers principles of human epicardium biology in heart development and disease." Nature Biotechnology 41.12 (2023): 1787-1800.

[7] Mutsaers, Steven E., et al. "Mesothelial cells in tissue repair and fibrosis." Frontiers in pharmacology 6 (2015): 113.

[8] Széplaki, Gábor, et al. "Association of high serum concentration of the third component of complement (C3) with pre-existing severe coronary artery disease and new vascular events in women." Atherosclerosis 177.2 (2004): 383-389.

[9] Waterworth, Dawn M., et al. "Genetic variants influencing circulating lipid levels and risk of coronary artery disease." Arteriosclerosis, thrombosis, and vascular biology 30.11 (2010): 2264-2276.

[10] Lake, Blue B., et al. "An atlas of healthy and injured cell states and niches in the human kidney." Nature 619.7970 (2023): 585-594.

[11] McEvoy, Caitriona M., et al. "Single-cell profiling of healthy human kidney reveals features of sex-based transcriptional programs and tissue-specific immunity." Nature communications 13.1 (2022): 7634.

[12] Xu, Hui, and Zenggeng Wang. "MicroRNA-23a-3p ameliorates acute kidney injury by targeting FKBP5 and NF-κB signaling in sepsis." Cytokine 155 (2022): 155898.

[13] Song, Anni, et al. "The role of CXCL12 in kidney diseases: a friend or foe?." Kidney Diseases 7.3 (2021): 176-185.

[14] Ziegler CG, Miao VN, Owings AH. et al. Impaired local intrinsic immunity to sars-cov-2 infection in severe covid-19. Cell 2021; 184:4713–4733.
